# HIF2α drives ccRCC metastasis through transcriptional activation of methylation-controlled J protein and enhanced prolegumain secretion

**DOI:** 10.1038/s41419-025-07432-3

**Published:** 2025-02-13

**Authors:** Tianyu Shen, Yu Su, Dekun Wang, Gang Li, Xuan Liu, Chuangxin Sun, Taoyu Hu, Haoxiang Pang, Xue Mi, Yuying Zhang, Shijing Yue, Zhujun Zhang, Xiaoyue Tan

**Affiliations:** 1 The School of Medicine, Nankai University; 94 Wei Jin Road, Tianjin, China; 2 Department of Urology, Tianjin Institute of Urology, the 2nd Hospital of Tianjin Medical University, 23 Ping Jiang Road, Tianjin, China

**Keywords:** Renal cell carcinoma, Cancer genetics

## Abstract

The role of hypoxia-inducible factor 2α (HIF2α) in clear cell Renal Cell Carcinoma (ccRCC) is still not fully understood. In this study, we identified that urinary prolegumain levels positively correlated with the malignant characteristics of ccRCC. In cultured 786-O and OSRC-2 cells, HIF2α downregulation reduced prolegumain secretion. RNA sequencing assay revealed that HIF2α induces methylation-controlled J (MCJ), a negative regulator on the mitochondrial respiratory chain. Silencing MCJ reduced prolegumain secretion, and MCJ overexpression restored prolegumain secretion inhibited by HIF2α downregulation. Chromatin immunoprecipitation and luciferase assay confirmed MCJ as a transcription target of HIF2α. Furthermore, we showed the ectopic MCJ overexpression reversed the improved mitochondrial damage resulting from HIF2α downregulation, as evidenced by electron microscope, ATP level, GSSG/GSH ratio, MitoSOX, and DHE staining. Through mass spectrometry analysis, we identified oxidation site His343 on the legumain sequence as contributing to the prolegumain secretion. Therapeutically, silencing MCJ or HIF2α or using ROS scavengers Vitamin C or MitoQ alleviated MMP2 activation as well as cell migration and tube formation. In a mouse orthotopic xenograft model of ccRCC, silencing MCJ or administration of MitoQ significantly protected against mitochondrial damage and subsequently reduced the lung metastasis of tumors. Overall, our study identified MCJ as a target molecule of HIF2α in ccRCC. Silencing MCJ or using ROS scavengers like MitoQ can suppress oxidation site His343 in legumain, preventing prolegumain secretion and subsequently reducing metastasis of ccRCC.

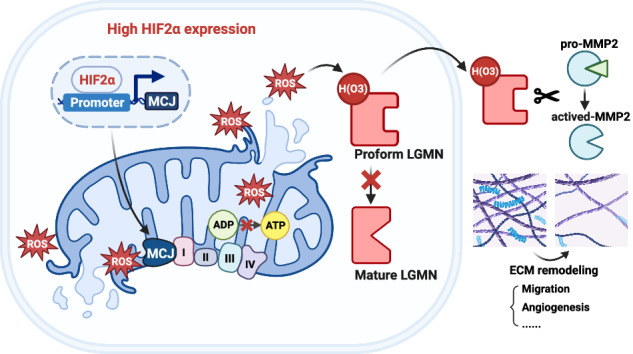

## Introduction

Renal cell carcinoma (RCC) arises from the epithelial cells of the nephron tubules and constitutes approximately 85% of all malignant renal tumors. Among the various histologic subtypes, clear cell renal cell carcinoma (ccRCC) is the most prevalent, accounting for 75% of cases [1]. While patients with localized ccRCC confined to the kidney enjoy high cure rates, the 5-year survival rate for those with metastatic RCC remains dismal [2]. There is an urgent need to identify novel therapeutic targets for the early detection and effective treatment of metastatic RCC.

The von Hippel-Lindau (VHL) tumor suppressor gene mutation is present in up to 90% of ccRCCs [3]. The VHL gene product, pVHL, directs the degradation of the α-subunit of hypoxia-inducible factor (HIF). When VHL is inactivated, HIFα accumulates and undergoes nuclear translocation [4, 5]. This activation of HIFα triggers the continuous transcription of downstream genes that are crucial for tumor growth and metastasis [6]. Growing evidence indicates that HIF1α and HIF2α exert distinct effects in the pathogenesis of ccRCC. HIF2α acts as an oncogene, while HIF1α functions as a tumor suppressor in ccRCC [7, 8]. Small molecule inhibitors that target HIF2α can halt the transcription of key genes associated with the development of ccRCC, demonstrating targeted antitumor activity in mouse xenograft models [9]. Despite the promising initial findings, the clinical application of HIF2α inhibitors encounters numerous challenges, and combination therapies are emerging as a promising strategy to pursue.

Legumain, also known as asparaginyl endopeptidase (AEP), is associated with the caspase family due to its approximately 15% sequence homology with caspases and its strict specificity at the P1 position [10, 11]. It has been reported that legumain exhibits a broad tissue distribution in mammals, with the highest concentration found in the kidney [12]. Legumain is predominantly activated from its precursor form through an autocatalytic process that occurs within the endo-lysosomal system. However, it is also found on the cell membrane and released extracellularly. These compartments are characterized by similar environmental conditions, including an acidic pH and a reducing redox potential [11, 13]. Overexpression of legumain has been observed in various cancer types and is correlated with increased malignancy and poor prognosis [14–17]. In tumor tissues, secreted prolegumain present in the tumor microenvironment can undergo autoactivation due to the acidic pH, thereby promoting metastasis through the remodeling of the extracellular matrix [18–21]. Therefore, understanding the mechanisms that regulate legumain secretion is crucial for the development of targeted therapeutic strategies.

Our findings reveal that HIF2α triggers mitochondrial impairment and reactive oxygen species (ROS) accumulation, which in turn enhances legumain secretion. The mitochondrial regulator MCJ plays a pivotal role in modulating HIF2α‘s impact on mitochondrial dysfunction within ccRCC cells. Suppression of MCJ or neutralization of ROS both reduce legumain secretion and, importantly, mitigate tumor metastasis in vivo.

## Materials and methods

### Animal experiments

A total of 24 six-week-old male BALB/c nude mice, sourced from Vital River, Beijing, China, were randomly assigned to four experimental groups, each comprising six mice. All mice had a weight range of 16–19 g and a length of 7–7.5 cm. We utilized 786-O cells stably expressing Luciferase to monitor tumor growth. A ccRCC mouse model was established by intrarenal injection of 1 ×10^6^ 786-O cells with stable MCJ silencing or scramble control. The MitoQ treatment group received MitoQ four weeks post-implantation, at a dosage of 5 mg/kg body weight administered twice weekly for a continuous three-week period. MitoQ was dissolved in normal saline, which also served as the vehicle control. Tumor growth and metastasis were tracked using a Small Animal Live Imager (IVIS Lumina II, Caliper Life Sciences). The endpoint for the animal experiments was set at seven weeks following the implantation of tumor cells. Mice were euthanized using CO_2_ at a flow rate of 35% per minute to minimize suffering. Subsequently, kidney and lung tissues were harvested, processed, and subjected to hematoxylin and eosin (H&E) staining, immunohistochemistry (IHC), and Western blot analysis. The above animal experiments were non-blind.

### Cell culture, cell treatment, and stable cell line establishment

The human renal proximal tubule epithelial cell line HK-2, ccRCC cell lines ACHN, 769-P, 786-O, Caki-1, OSRC-2, and the human umbilical vein endothelial cell line HUVEC were all procured from the American Type Culture Collection (ATCC). They were recently authenticated by STR profiling and tested for mycoplasma contamination. These cells were maintained in either RPMI-1640 or DMEM (Gibco, Massachusetts, USA), supplemented with 10% fetal bovine serum (Gibco), and incubated at 37 °C in a humidified atmosphere containing 5% CO_2_. For transfection, lentivirus from GeneChem (Shanghai, China) was utilized to encapsulate plasmids. Following transduction, cells were selected using puromycin (5 μg/ml) to isolate those with stable knockdown of HIF1α, HIF2α, Legumain, MCJ, overexpression of MCJ, and stable luciferase expression, respectively. The target sequences for shRNA were as follows: *HIF1α*: CCGCTGGAGACACAATCATAT; *HIF2α*: GCGCAAATGTACCCAATGATA; *LGMN*: GTATTGAGAAGGGTCATATTT; *DNAJC15* (*MCJ*): GCAAAAGACTTGCTAGAAA.

### Human samples

The human kidney tissue samples were collected from patients with clear cell renal cell carcinoma (ccRCC) who underwent partial nephrectomy at the Second Affiliated Hospital of Tianjin Medical University. A total of 16 pairs of adjacent non-tumor and tumor tissues were surgically excised. A portion of each sample was embedded in paraffin for subsequent IHC analysis, while the remaining tissue was snap-frozen in liquid nitrogen for further western blotting and qRT-PCR assessments. Additionally, blood and urine samples (*n* = 100) were collected from both ccRCC patients and individuals without tumors for the detection of legumain through ELISA and western blot.

### Antibodies

The antibodies utilized in this study for both western blotting and IHC assays are as follows: LGMN (catalog number ab183028, diluted at 1:10000 for WB and 1:2000 for IHC), and MMP2 (catalog number ab92536, diluted at 1:1000 for WB and 1:200 for IHC) were sourced from Abcam (Cambridge, MA, USA). HIF2α (catalog number NB100-122, diluted at 1:1000 for WB and 1:200 for IHC), and HIF1α (catalog number NB100-105, diluted at 1:1000 for WB and 1:100 for IHC) were procured from Novus (Littleton, CO, USA). MCJ (catalog number 16063-1-AP, diluted at 1:1000 for WB and 1:200 for IHC), and β-actin (catalog number 20536-1-AP, diluted at 1:5000 for WB) were obtained from Proteintech (Chicago, IL, USA). For WB, Goat anti-Mouse/Rabbit Secondary Antibody (catalog numbers 31430/31460, both diluted at 1:10000) were used and were purchased from Thermo Fisher Scientific (Waltham, MA, USA).

### Western blot

Cells were lysed using RIPA buffer supplemented with phenylmethylsulphonyl fluoride to extract total protein. Protein concentration was determined using the BCA assay. Subsequently, proteins were resolved by sodium dodecyl sulfate-polyacrylamide gel electrophoresis. Following electrophoresis, the proteins were transferred onto a polyvinylidene fluoride (PVDF) membrane at a constant current of 250 mA for 120 min. The PVDF membrane was then blocked with non-fat dry milk and incubated with the primary antibody overnight at 4 °C. Afterward, the membrane was incubated with the appropriate secondary antibody at room temperature for 1 h. Finally, protein bands were visualized using a Western blotting detection system (Tanon 4500, Shanghai, China).

### Hematoxylin-eosin (HE) staining

Paraffin-embedded tissue sections were subjected to deparaffinization. The nuclei were stained with hematoxylin for a duration of 2 min, followed by differentiation using a 0.5% hydrochloric acid solution in alcohol. The sections were then rinsed under tap water for 5 min to remove excess stains. Subsequently, the cytoplasm was stained with eosin. The dehydration process was carried out and then the sections were mounted with neutral gum.

### Immunohistochemistry (IHC) staining

Paraffin-embedded tissue sections underwent deparaffinization and antigen retrieval procedures. Endogenous peroxidase activity was quenched using 3% H_2_O_2_. Tissues were closed with 5% normal goat serum for 2 h, followed by primary antibody incubation, overnight at 4 °C. After removing the primary antibody, the sections were then incubated with the secondary antibody at room temperature for 2 h. The DAB staining solution was applied dropwise to develop the color. The color development was halted by immersing the slides in tap water, and the nuclei were subsequently counterstained with hematoxylin. The sections were then dehydrated through a series of alcohol washes and permanently sealed with neutral gum. For quantitative analysis, the staining intensity was graded as follows: 0 for negative or weak staining, 1 for moderate staining, and 2 for strong staining. The distribution area of staining was also scored: 0 for less than 5% positive staining, 1 for 5% to 50% positive staining, and 2 for more than 50% positive staining. The final H-score was calculated as the sum of the staining intensity and distribution area scores.

### Quantitative real-time PCR (qRT-PCR) analysis

Total RNA was isolated from the samples using TRIzol reagent (15596026, Thermo Fisher Scientific, Inc., MA, USA). The purified RNA was subsequently reverse-transcribed into cDNA. qRT-PCR was conducted using the synthesized cDNA as a template. β-actin served as an endogenous control to normalize the gene expression data. Relative gene expression levels were quantified using the 2^-ΔΔCt method. The following primer sequences were used for amplification: HIF1α forward primer5′-gaacgtcgaaaagaaaagtctcg-3′, reverse primer 5′-ccttatcaagatgcgaactcaca-3′; HIF2α forward primer 5′-cggaggtgttctatgagctgg-3′, reverse primer 5′-agcttgtgtgttcgcaggaaa-3′; Legumain forward primer 5′-atcgtggcaggttcaaatgg-3′, reverse primer 5′-ggactccctgatagacatctgtg-3′; MCJ forward primer 5′-ttgcaggtcgctacgcattt-3′, reverse primer 5′-ccagcttctcgcctactcattt-3′.

### Chromatin immunoprecipitation (ChIP)

Primers were designed for different regions of the MCJ promoter. The interaction was determined by ChIP assay on prepared cells using the EpiQuik ChIP Kit (Epigentek, Farmingdale, NY, USA) following the protocol provided. In brief, DNA fragments that were immunoprecipitated with an antibody against HIF2α were subjected to PCR analysis. The PCR was carried out using the following primer pairs: CHIP1(-1500 bp ~ -1200 bp) forward primer 5’-ccaggaatgtacaagacagcttgga-3’ and reverse primer 5’- gctttattttttttctattcctgaa-3’; CHIP2 (-1200 bp ~ -900 bp) forward primer 5’-tttttcttcctgtcagctgattttg-3’ and reverse primer 5’-gtacctgggattaaaggcacgtgcc-3’; CHIP3 (-900 ~ -600 bp) forward primer 5’-tcgggaggcagagggtgcagtgagc-3’ and reverse primer 5’- ggatcacaccagcttggccgacatg-3’; CHIP4 (-600 ~ -300 bp) forward primer 5’-acccgcctcggtctcccaaagtgct-3’ and reverse primer 5’-gttggtcgaatcagatgactgccca-3’; CHIP5 (-300 ~ -1 bp) forward primer 5’-atcagttcgcagggcttaagcccag-3, and reverse primer 5’-acgaggcggccgtagggacaaacta-3.

In addition, potential binding sites of HIF2α within the MCJ promoter region were identified using the online tool JASPAR [22] (https://jaspar.genereg.net/). The primer was designed again based on the predicted binding region and validated using ChIP assay. The PCR was carried out using the following primer pairs: CHIP (-925bp ~ -917 bp) forward primer 5’-tcaggagtttgagaccagcctggcc-3’ and reverse primer 5’-cctgggattaaaggcacgtgccacc-3’;

### Luciferase reporter assays

Luciferase reporter plasmids, specifically the MCS-firefly luciferase and TK promoter-Renilla luciferase, were obtained through GeneChem (Shanghai) Co., Ltd., China. The 293T cells were transfected with the MCJ luciferase reporter construct containing the MCS-firefly luciferase sequence, either alone or in combination with a plasmid overexpressing HIF2α. To normalize transfection efficiency and cell number, luciferase activity was measured relative to the activity of Renilla luciferase.

### GEO dataset analysis

The gene expression profiles GSE115389 [23] from the NCBI GEO database (http://www.ncbi.nlm.nih.gov/geo) were downloaded. Probe-level information was mapped to their corresponding genetic symbols using interpretation data also obtained from the GEO platform. A volcano plot was generated to visualize the consistent differentially expressed genes (DEGs). The R language’s Hclust function was utilized for the analysis of these differential genes, with a threshold set at |log_2_FC | ≥1.0 and *P*-value ≤ 0.05 to determine statistical significance.

### Intracellular and mitochondrial ROS detection

The intracellular and mitochondrial ROS were measured using dihydroethidium (DHE) (0063, Beyotime, Shanghai, China) fluorescent probes and MitoSOX™ Red (M36008; Thermo Fisher Scientific, Inc., MA, USA). To assess ROS, cells were treated with 10 µM DHE or 5 µM MitoSOX™ Red and incubated for 30 min at 37 °C in a dark environment. The cells were visualized using a fluorescence microscope (Vert. A1, Zeiss, Germany).

### Measurement of GSH/GSSG ratio and ATP quantification

The levels of reduced glutathione (GSH) and oxidized glutathione (GSSG) were quantified using the GSH Content Assay Kit (catalog number BC1175, Solarbio, Beijing, Chin) and the GSSG Content Assay Kit (catalog number BC1185, Solarbio). These assays were conducted by the protocols provided by the manufacturer. Additionally, ATP levels were measured using a colorimetric ATP content assay kit (catalog number BC0300, Solarbio). This assay was performed following the detailed instructions issued by the manufacturer.

### LC-MS modification identification

After completing the protein electrophoresis, the SDS gel was stained with Coomassie Blue to visualize the proteins. The gel was then cut based on the molecular weight markers, and the slices containing the proteins of interest were excised and prepared for mass spectrometry analysis. The prepared samples were loaded onto Zorbax 300SB-C18 peptide traps (Agilent Technologies, Delaware, USA). The enzymatic digestion products were then separated by capillary HPLC, and the separated peptides were introduced into a Q Exactive HF-X mass spectrometer (Thermo Fisher Scientific, Massachusetts, USA) for mass spectrometry analysis. During the analysis, the mass-to-charge (m/z) ratios of the peptides and their fragments were determined through full-scan mass spectrometry. The raw data files generated by the mass spectrometry assays were processed using the MaxQuant software (Max-Planck-Institute of Biochemistry, Germany).

### Transwell invasion assay

500 µL of 1640 medium, supplemented with 10% FBS, was added to the bottom chamber of the transwell plate. Then, 200 µL of a cell suspension, prepared to a concentration of 3 × 10^5^ cells/mL, was gently pipetted into the upper chamber. The assembly was incubated at 37 °C for 24 h to facilitate cell migration. After the incubation period, the medium from both chambers was removed. The cells were fixed with 4% paraformaldehyde to preserve their structure, followed by staining with crystal violet to visualize the migrated cells. The upper side of the membrane was cleaned with a cotton swab to remove any unattached cells. Microscopic images of the cells on the lower surface of the membrane were captured using a microscope. These images were then imported into ImageJ software for quantitative analysis.

### Tube formation assay

Matrigel was thawed overnight at 4 °C the day before the experiment. After adding 50 μl of matrigel to each well of a 96-well plate, the plate was placed in a 37 °C, 5% CO_2_ incubator for 30 min to allow the gel to solidify. Suspend HUVEC cells using the conditioned medium for tumor cells, and add 100 μl of cell suspension containing 2 × 10^4^ HUVEC cells per well. After 6 hours of incubation at 37 °C and 5% CO_2_, the cells were photographed and the number of completed capillary-like structures was counted under the microscope.

### Enzyme-linked immunosorbent assay (ELISA)

LGMN concentrations in serum and urine were measured using a human LGMN ELISA kit (MM-52689H1, MEIMIAN, Jiangsu, China) following the manufacturer’s instructions. Briefly, add sample 50 μl to the 100 μl HRP-conjugate reagent, then incubate for 60 min at 37 °C. Discard the Liquid, dry by swing, add washing buffer to every well, still for 30 s then drain, repeat 5 times. Add Chromogen Solution A 50 ul and Chromogen Solution B to each well, and evade the light preservation for 15 min at 37 °C. Read absorbance at 450 nm using a enzyme-labeled instrument (Multiskan FC, Thermo Fisher Scientific, Inc., MA, USA).

### Statistical analysis

Statistical analysis was conducted using SPSS 20.0 statistical software (IBM Corp.). Each experiment in this study was independently repeated at least three times. The data are presented as the mean ± SD. To compare the results across different groups, a one-way analysis of variance (ANOVA) was employed. When direct comparisons between two groups were necessary, an independent sample unpaired Student’s *t* test or paired *t*-test was utilized. To quantify the linear relationship between two continuous variables, Pearson correlation analysis was performed. For examining the relationship between categorical variables, a chi-square test was applied. A statistically significant difference was considered to exist when the *P*-value was less than 0.05. * for *P* < 0.05, ** for *P* < 0.01, and *** for *P* < 0.001, indicating increasing levels of statistical significance.

## Results

### Urinary levels of legumain correlate positively with the metastatic state of ccRCC

**Fig. 1:**
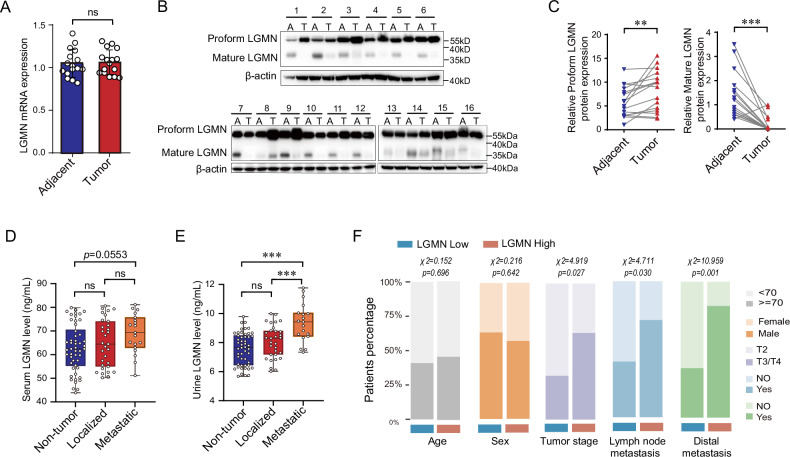
Urinary levels of legumain correlate positively with metastatic state of ccRCC. Tumor tissues of ccRCC and adjacent kidney tissues were collected (*n* = 16). **A** mRNA level of *Legumain* in the tissue homogenates detected by qRT-PCR. **B** Representative image of Western blot using antibody against Legumain for 16 pairs of samples. A, adjacent tissue; T, tumor tissue. **C** Statistical analysis on the all 16 pairs of samples using paired *t*-test. Peripheral blood and urine samples were collected from the non-tumor individuals, patients with localized ccRCC, and patients with metastatic ccRCC, respectively. **D** ELISA analysis of the Legumain concentration in the serum. Each dot represents one patient sample. **E** ELISA analysis of legumain in the urine samples. Each dot represents one patient sample. **F** The correlation between urinary level of legumain and clinicopathologic features was analyzed using a Chi-square test. Data represented as mean ± SD. *P < 0.05; **P < 0.01; ***P < 0.001.

Previous research has established a positive correlation between legumain and the malignant characteristics of various tumors. In our study, we collected 16 pairs of tumor and adjacent kidney tissues from patients diagnosed with ccRCC and compared the expression of legumain at both the mRNA and protein levels. Quantitative Real-time PCR indicated no significant difference in *Lgmn* mRNA levels between the ccRCC tumor and adjacent kidney tissues (Fig. 1A). In contrast, immunoblotting revealed an increase in prolegumain and a decrease in mature legumain in tumor tissues compared to adjacent kidney tissues (Fig. 1B, C). The activity and stability of legumain are highly dependent on the pH of the environment, and the stability of intracellular prolegumain is known to promote the secretion of legumain. We proceeded to measure the levels of prolegumain in serum and urine samples from 50 healthy controls, 31 patients with localized ccRCC, and 19 patients with metastatic ccRCC. ELISA results showed no significant difference in serum legumain levels among healthy controls and patients with localized or metastatic ccRCC, although a trend towards an increase was observed (Fig. 1D). However, the urinary level of legumain was significantly higher in patients with metastatic ccRCC compared to those with localized tumors or healthy controls (Fig. 1E). We utilized chi-square analysis to investigate the relationship between the urinary level of legumain and various categorical variables associated with ccRCC. The findings revealed a positive correlation between elevated urinary legumain levels and disease stage, as well as the frequency of lymph node or distal metastasis (Fig. 1F). Thus, our data suggest that the secretion of legumain could serve as a potential biomarker for the metastasis of ccRCC.

### HIF2α promotes the extracellular secretion of prolegumain in ccRCC cells

**Fig. 2:**
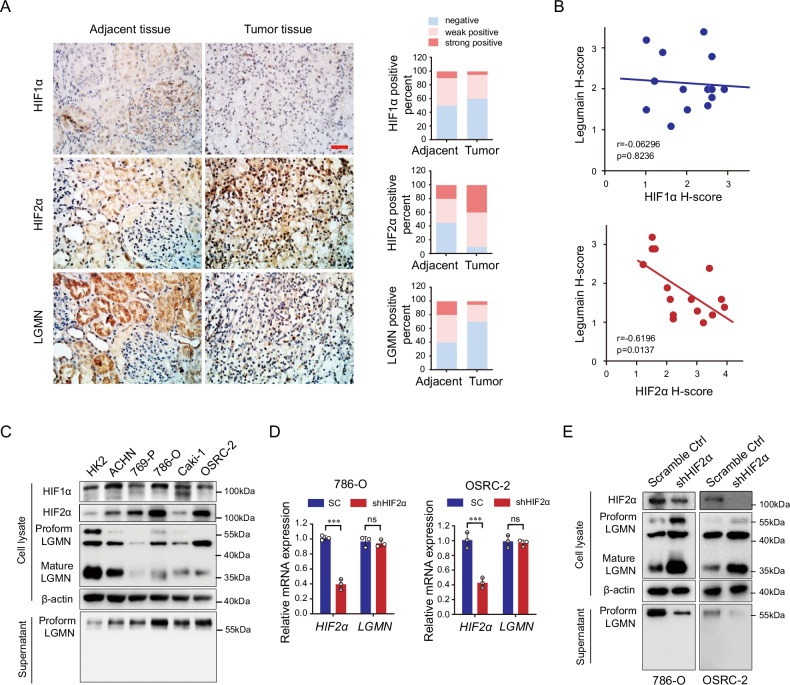
HIF2α induces the secretion of legumain in renal cell carcinoma cells. ccRCC tumor tissues and adjacent kidney tissues were collected (*n* = 16). IHC staining was conducted on the tissue sections using antibodies against HIF1α, HIF2α, and legumain. **A** The left panel displays representative IHC images, and the right panel presents the statistical analysis of staining intensity. Scale bar: 50 μm. **B** Linear correlation analysis was conducted on the expression of legumain with HIF1α or HIF2α using the Pearson test. **C** Western blot analysis was performed using antibodies against legumain, HIF1α, and HIF2α in cell lysate or supernatant from ccRCC cell lines and control HK2 cells as indicated. Stable HIF2α-knocking down 786-O and OSRC-2 cell lines were established. **D** mRNA expression levels of *LGMN* and *HIF2α* were determined using qRT-PCR. **E** Immunoblot was conducted using antibodies against legumain and HIF2α. Data are presented as mean ± SD. **P < 0.01; ***P < 0.001.

It is recognized that the transcription factors HIF1α and HIF2α are pivotal in driving the initiation and progression of ccRCC. Our interest lies in exploring whether HIFs are involved in the secretion of prolegumain. IHC staining revealed a marked increase and nuclear translocation of HIF2α in ccRCC tumor tissues. Legumain expression, primarily localized in tubular cells, was found to be diminished in tumor tissues (Fig. 2A). Correlation analysis indicated an inverse relationship between HIF2α levels and legumain, whereas no significant association was observed between HIF1α and legumain (Fig. 2B). We further examined the expression of HIFs and legumain across different ccRCC cell lines. A pronounced upregulation of HIF2α was noted in renal cell carcinoma cells in comparison to normal tubular cells. Immunoblotting results showed a reduction of both pro- (approximately 55 kDa) and mature legumain (approximately 32 kDa) in the cell lysates of tumor cells. Conversely, prolegumain levels were increased in the supernatant from tumor cell lines compared to those from control tubular cells (Fig. 2C). To assess the impact of HIF2α on the distribution of legumain, we established stable cell lines with HIF2α silenced in 786-O or OSRC-2 cells. Real-time PCR indicated that the downregulation of HIF2α did not influence the mRNA expression of legumain (Fig. 2D). Immunoblotting demonstrated that the reduction of HIF2α led to an increase in the intracellular levels of legumain and a decrease in the levels of prolegumain in the supernatant from 786-O or OSRC-2 cells (Fig. 2E). In conclusion, our findings suggest that HIF2α facilitates the secretion of legumain in ccRCC.

### MCJ mediates the inductive effect of HIF2α on Legumain secretion

**Fig. 3:**
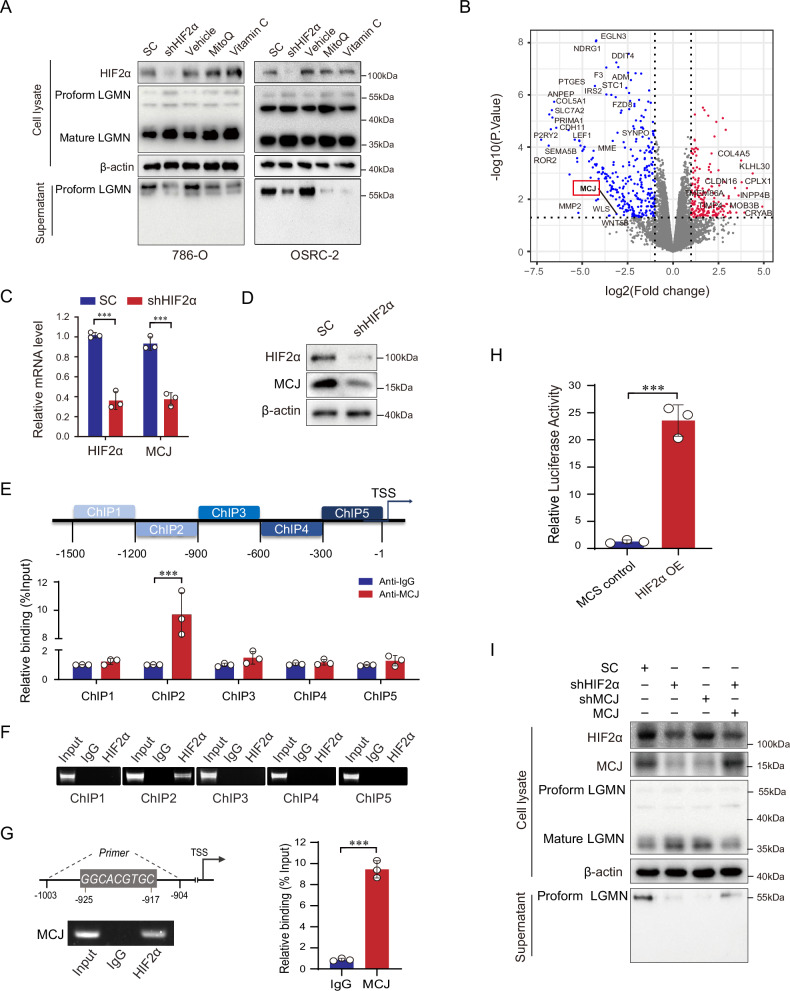
MCJ mediates the inductive effect of HIF2α on Legumain secretion. Stable HIF2α- and MCJ-knocking down 786-O and OSRC-2 cell lines were established, and these cells were treated with control group solvent, MitoQ, or Vitamin C, respectively. **A** Immunoblot was conducted using an antibody against legumain in the cell lysate or supernatant. The GSE11538 dataset comprises 2 wild-type groups and 3 HIF2α knockout groups. Cluster analysis was performed based on differentially expressed genes. **B** The volcano plot displayed differentially expressed genes with |log2FC | ≥ 1.0, *P*-value ≤ 0.05. **C** MCJ mRNA expression was detected by qRT-PCR, and **D** immunoblot was conducted using an antibody against MCJ in cell lysate from stable HIF2α-silencing and scramble control 786-O and OSRC-2 cell lines. **E**, **F** ChIP-qPCR confirmed one binding site of HIF2α in the promoter of the MCJ gene. Strip diagrams from electrophoretic gels based on ChIP-qPCR products confirmed this result. **G** ChIP-qPCR validated the binding domains predicted by JASPAR. Strip diagrams from electrophoretic gels based on ChIP-qPCR products confirmed this result. **H** A luciferase reporter assay was used to measure MCJ promoter activity. Stable HIF2α- and MCJ-knocking down 786-O cell lines were established, and MCJ was transiently overexpressed in the stable HIF2α-knocking down 786-O cells. **I** Immunoblot was performed in cell lysate and supernatant using an antibody against Legumain. Data are represented as mean ± SD. ***P < 0.001.

Oxidation sites that are responsive to ROS have been reported to regulate the processing of legumain [18]. We then investigated whether a reduction in ROS influences the secretion of legumain. The effects of vitamin C or MitoQ on cell viability (Supplementary Fig. 1A, B) and their efficiency in reducing ROS (Supplementary Fig. 1C–F) were examined separately. In the 786-O and OSRC-2 cell lines, the administration of vitamin C or MitoQ significantly inhibited the secretion of prolegumain (Fig. 3A). To identify the downstream target gene of HIF2α responsible for the excess ROS, we performed a differential gene analysis using dataset GSE115389 [23] from the GEO database. This dataset performed RNA-Seq analysis of wildtype and three HIF2α knockout 786-O single-cell clones generated by CRISPR/Cas9 to identify the HIF2α-responsive genes in the cell line. We observed that *MCJ*, a negative regulator of mitochondrial function, was among the genes that were significantly downregulated following the knockout of HIF2α in 786-O cells (Fig. 3B). A positive correlation was observed between HIF2α and MCJ in ccRCCs when analyzing the TCGA database (Supplementary Fig. 2). Quantitative real-time PCR and immunoblotting confirmed the downregulation of MCJ after the silencing of HIF2α in 786-O cells (Fig. 3C, D). ChIP-qPCR assay demonstrated that HIF2α binds to the promoter region of the *MCJ* gene at approximately −1200 to −900 base pairs upstream of the transcription start site (TSS) (Fig. 3E), which was qualitatively confirmed by agarose gel electrophoresis (Fig. 3F). JASPAR prediction showed that the promoter region of HIF2α and MCJ gene is located at −925 to −917 base pairs upstream of TSS. This binding domain was validated by ChIP-qPCR, which was qualitatively confirmed by agarose gel electrophoresis (Fig. 3G). A luciferase assay showed that the transcriptional activity of the *MCJ* gene was significantly increased upon overexpression of HIF2α (Fig. 3H). All these data support that MCJ is a downstream target gene under the transcriptional regulation of HIF2α. Furthermore, we found that silencing MCJ mimicked the inhibitory effect of HIF2α downregulation on the secretion of legumain, and this effect could be rescued by ectopic overexpression of MCJ (Fig. 3I). In summary, our results suggest that HIF2α transcriptionally upregulates MCJ, which in turn leads to the secretion of legumain.

### Activation of HIF2α/MCJ leads to excess ROS accumulation

**Fig. 4:**
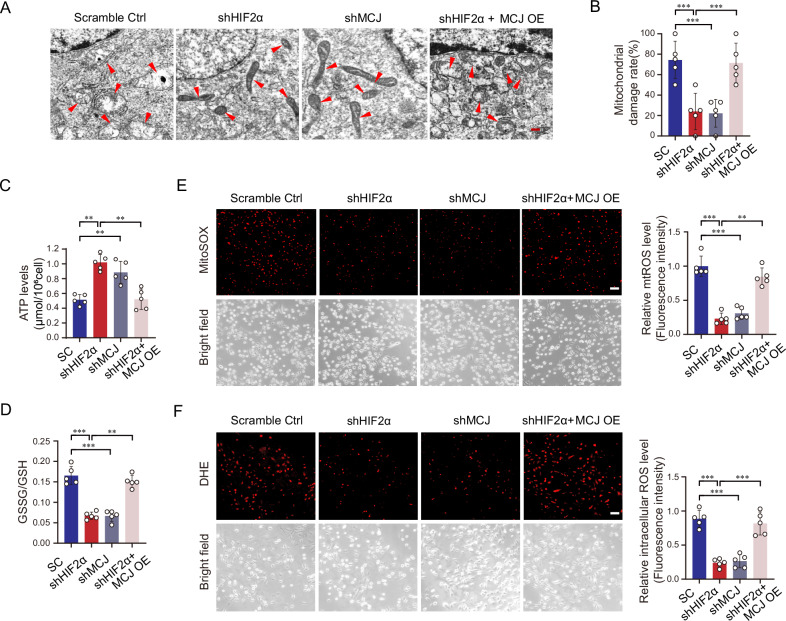
Activation of HIF2α/MCJ leads to excess ROS accumulation. Stable HIF2α and MCJ knockdown 786-O cell lines were established, and MCJ was transiently overexpressed in the stable HIF2α knockdown 786-O cells. **A** Representative images of transmission electron microscopy are shown. Red arrows indicate the mitochondria. Scale bar: 100 nm. **B** Statistical analysis of the percentage of damaged mitochondria is presented. **C**, **D** The concentrations of cellular ATP and the GSSG/GSH ratio are measured. **E** The amount of mitochondrial ROS was detected using MitoSOX probes. **F** Total intracellular ROS were measured using the DHE probe. The left panel displays representative images, while the right panel presents the statistical analysis results. Scale bar: 50 μm. Data are presented as the mean ± SD. **P < 0.01; ***P < 0.001.

MCJ acts as a negative regulator of mitochondrial function, exerting its effects by binding to complex I of the mitochondrial respiratory chain [24, 25]. As shown by transmission electron microscopy, signs of mitochondrial damage, such as swelling of the mitochondria and irregularities, distortions, fractures, and loss of cristae, were evident in the control 786-O cells. The silencing of HIF2α or MCJ mitigated these signs of mitochondrial injury, whereas the overexpression of MCJ negated the protective effect of HIF2α silencing on mitochondrial health (Fig. 4A, B). The level of ATP is indicative of the functional state of mitochondria. Our findings revealed that the downregulation of HIF2α or MCJ increased the ATP levels, while the overexpression of MCJ restored the ATP levels that had risen due to the silencing of HIF2α (Fig. 4C). The ratio of GSSG to GSH reflects the redox equilibrium. Consistent with the ATP results, the silencing of HIF2α or MCJ decreased the oxidation of GSH, and the overexpression of MCJ reversed the redox balance disrupted by HIF2α silencing (Fig. 4D). To specifically label mitochondrial and intracellular ROS, we employed MitoSOX and DHE, respectively. Fluorescence staining indicated that both mitochondrial and intracellular ROS levels were significantly increased when HIF2α or MCJ was knocked down. Furthermore, the ectopic overexpression of MCJ counteracted the rise in ROS levels caused by the knockdown of HIF2α (Fig. 4E, F). In summary, our data suggest that MCJ, induced by HIF2α, plays a role in promoting oxidative stress and the excessive accumulation of ROS.

### The oxidation site at position His343 facilitates the secretion of legumain in ccRCC

**Fig. 5:**
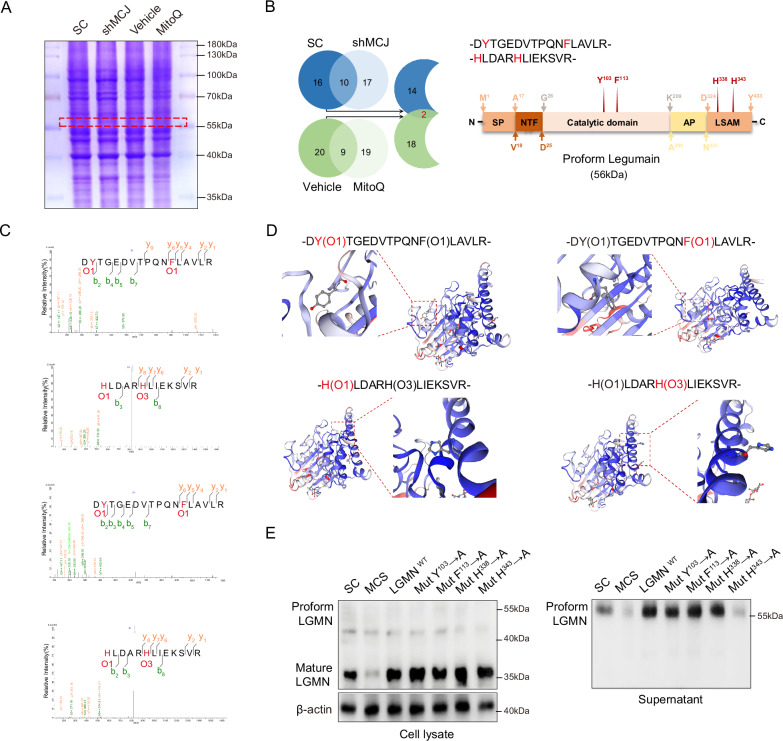
His343 Oxidation promotes secretion of prolegumain in ccRCC. Stable MCJ knockdown and scramble 786-O cell lines were established, and 786-O cells were treated with MitoQ or solvent control, respectively. **A** SDS-PAGE electrophoresis was performed on the total cell lysate, followed by staining with Coomassie Brilliant Blue. The red box indicates the location of the excised gel piece subjected to LC-MS analysis. **B** The left panel provides a schematic representation of the process for screening candidate oxidized sites. The right panel indicates the positions of oxidized residues within the legumain amino acid sequence. **C** Characteristics of the identified differentially oxidized sites by LC-MS are presented. O1: one oxygen atom added; O3: three oxygen atoms added. **D** Cartoon models illustrate the amino acid residues bearing the differentially oxidized sites. Legumain overexpression plasmids harboring the mutations Y103A, F113A, H338A, and H343A were individually constructed and transfected into 786-O cells with legumain knockdown. **E** Immunoblot was conducted using an anti-legumain antibody in the cell lysate and supernatant.

Given the evidence that activation of HIF2α/MCJ leads to excess ROS accumulation, we further investigated whether an oxidation site regulates the processing of legumain under these conditions. Gel electrophoresis was conducted on cell lysates from the scramble control, the stable MCJ-knocking down 786-O cells, and wild type 786-O cells treated with either DMSO or MitoQ. We focused on bands corresponding to the molecular weight of prolegumain (56 kDa), and prepared protein samples for mass spectrometry analysis (Fig. 5A). Mass spectrometry analysis identified 16 differentially oxidized peptides in the stable MCJ-knocking down versus the scramble control 786-O cells, and 20 differentially oxidized peptides in wild type 786-O cells treated with DMSO versus MitoQ. There was an overlap of two differentially oxidized peptides (Fig. 5B). The differential oxidation sites and their types were Tyr103 (O1), Val113 (O1), His338 (O1), and His343 (O3) (Fig. 5C, D). To confirm the role of oxidation sites in the secretion of legumain, we created individual mutants for the aforementioned oxidation sites and overexpressed them in 786-O cells. Immunoblotting data revealed that only the His343 mutant inhibited the secretion of legumain. Overall, these results suggest that the secretion of legumain is dependent on the oxidation site at His343 (Fig. 5E). In conclusion, we have demonstrated that the oxidation site at His343 mediates the secretion of legumain induced by the excess accumulation of ROS.

### Secreted legumain activates MMP2 and promotes tumor invasion and angiogenesis

**Fig. 6:**
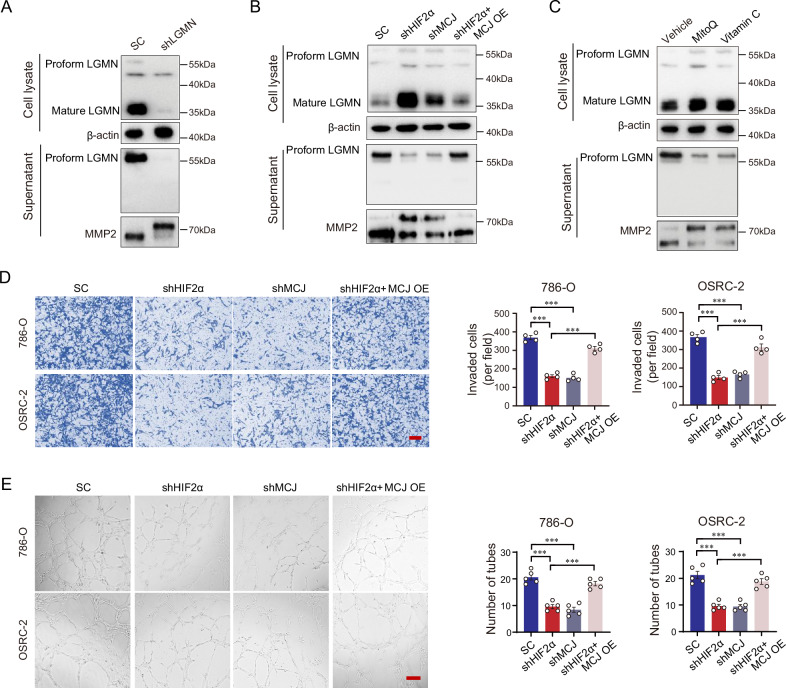
Secreted legumain activates MMP2 and promotes tumor invasion and angiogenesis. A stable legumain knockdown and a scramble control 786-O cell line were established. **A** Immunoblot was conducted using antibodies against legumain and MMP2. Stable HIF2α and MCJ knockdown 786-O cell lines were established, and MCJ was transiently overexpressed in the stable HIF2α knockdown 786-O cells. **B** Immunoblot was performed using antibodies against legumain and MMP2. Stable cell lines were established by knocking down HIF2α, MCJ, and subsequently rescuing MCJ after HIF2α knockdown in 786-O cells, respectively. **C** Immunoblot was conducted using antibodies against legumain and MMP2. 786-O cells were treated with MitoQ or Vitamin C, respectively. Stable HIF2α and MCJ knockdown 786-O and OSRC-2 cell lines were established, and MCJ was transiently overexpressed in the stable HIF2α knockdown 786-O cells. **D** Cell invasion capacity was assessed using the transwell assay. The left panel displays representative images, and the right panel presents the statistical analysis of the cell invasion ratio. **E** Angiogenesis was assessed using the tube formation assay. The left panel displays representative images, and the right panel presents the statistical analysis of the numbers of capillary-like structures. Five independent fields of view were taken for analysis. Scale bar: 50 μm. Data are presented as the mean ± SD. ***P < 0.001.

The acidic pH of the tumor microenvironment facilitates the self-processing and maturation of legumain, enabling it to exert its asparagine endopeptidase activity. We further explored the pro-metastatic effects of legumain induced through the activation of the HIF2α/MCJ pathway. As depicted in Fig. 6A, the silencing of legumain blocked the cleavage and maturation of pro-MMP2 (approximately 72 kDa) into its active form (approximately 62 kDa). The silencing of HIF2α or MCJ restored the cleavage of MMP-2, while the overexpression of MCJ counteracted the effect caused by the downregulation of HIF2α (Fig. 6B). Consistently, the ROS scavengers MitoQ and Vitamin C also inhibited the cleavage and maturation of MMP2 (Fig. 6C). An invasion assay was conducted to assess the invasive capacity of tumor cells. Our findings indicated that in both 786-O and OSRC-2 cells, the silencing of HIF2α or MCJ led to a decrease in tumor cell invasion, and the overexpression of MCJ negated the effect of HIF2α downregulation (Fig. 6D). We collected conditioned medium from 786-O or OSRC-2 cells and used it to stimulate the endothelial cell line HUVEC. The results demonstrated that the silencing of HIF2α or MCJ mitigated the tube formation induced by the conditioned medium from ccRCC cells, and this effect could be rescued by the overexpression of MCJ (Fig. 6E), without affecting the viability of HUVEC (Supplementary Fig. 3A, B).

**Fig. 7:**
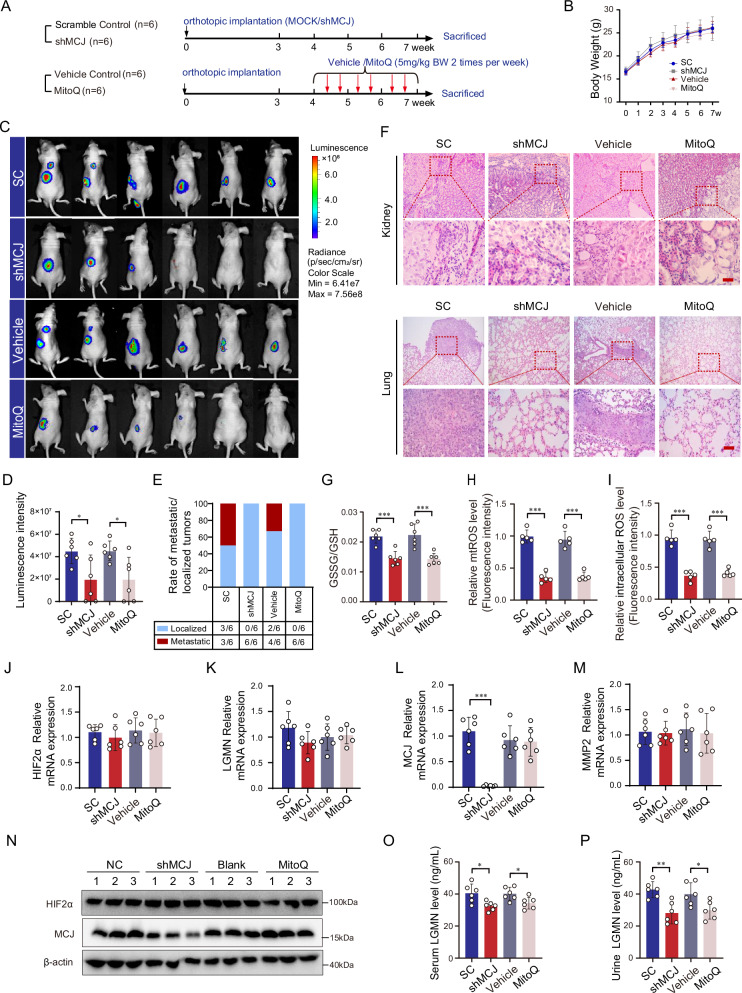
Inhibition of MCJ or scavenge of ROS alleviates legumain secretion and reduces metastasis of ccRCC in vivo. An in situ xenograft mouse model of ccRCC was established using 786-O cells stably overexpressing luciferase with either MCJ silenced or a scramble control. Additionally, MitoQ was administered to the ccRCC mouse model to examine the effect of a ROS scavenger (*n* = 6). **A** A flowchart of the in vivo experiments is provided. **B** Results from the statistical analysis on the body weight of animals are presented. **C** Images from the in vivo live luminescence imaging assay are shown. **D** Statistical analysis of the tumor luminescence intensity is provided. **E** The ratio of animals with metastatic versus localized tumors is shown. **F** Representative H&E staining images of kidney and lung tissue sections are displayed. Scale bar: 50 μm. Kidney tissue homogenates were isolated. **G** The GSSG/GSH ratio was determined. **H** Mitochondrial ROS were detected using the miSOX probe. **I** Intracellular ROS were measured using the DHE probe. **J**–**M** HIF2α, Legumain, MCJ and MMP2 mRNA expression were quantified by qRT-PCR. **N** HIF2α and MCJ protein expression were quantified by immunoblot. Blood and urine samples were collected. **L**, **M** Serum and urinary levels of legumain were measured using an ELISA. Data are presented as the mean ± SD. *P < 0.05; **P < 0.01; ***P < 0.001.

In vivo, an in situ xenograft ccRCC model was established using 786-O cells with stable MCJ silencing or scramble control. Additionally, MitoQ was administered to the ccRCC mouse model to evaluate the effects of a ROS scavenger (Fig. 7A). There was no significant difference in body weight among the treatment groups (Fig. 7B). Tumor size and metastatic state were assessed based on the intensity of luciferase fluorescence (Fig. 7C–F). Our findings indicated that silencing MCJ or administering MitoQ suppressed tumor growth and reduced lung metastasis in ccRCC (Fig. 7C–F). The redox status and ROS levels in tumor homogenates were also examined. The GSSG/GSH ratio, as well as the levels of mitochondrial (mtROS) and intracellular ROS, were significantly lower in the MCJ silencing and MitoQ groups compared to their respective controls (Fig. 7G–I). Knockdown of MCJ or administration of MitoQ did not affect the transcriptional level of the HIF2α, legumain, and MMP2 gene (Fig. 7J–M). Protein levels assayed in mouse kidney tissue illustrate a similar point (Fig. 7N). However, reduced levels of Legumain in serum and urine were observed in both the MCJ silencing and MitoQ groups, suggesting a change in legumain secretion due to the downregulation of MCJ or the scavenging of ROS (Fig. 7O, P). These results suggest that modulating the redox environment and ROS levels through MCJ silencing or ROS scavenging with MitoQ can significantly impact ccRCC progression and metastasis, potentially by affecting the secretion of legumain.

**Fig. 8:**
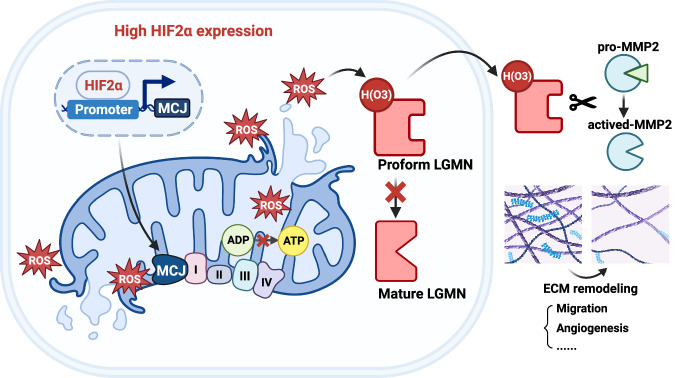
Schematic diagram of the effect of HIF2α-MCJ on the secretion of legumain and subsequently promoting tumor metastasis of ccRCC. HIF2α transcriptionally activates MCJ, leading to mitochondrial damage. The excessive accumulation of mtROS enhances the oxidation sites and secretion of prolegumain. Once extracellular, legumain influences the activation of MMP2, tumor invasion capacity, and angiogenesis, thereby playing a pro-metastatic role in ccRCC.

Overall, our findings suggest that legumain secretion, induced by HIF2α, contributes to the malignant characteristics of clear cell renal cell carcinoma (ccRCC). The activation of the HIF2α/MCJ pathway leads to an excessive accumulation of mitochondrial reactive oxygen species (mtROS), which in turn promotes the secretion of legumain through a specific oxidation site (Fig. 8). Consequently, the reduction of ROS through the silencing of MCJ or the use of ROS scavengers effectively impedes the progression of ccRCC.

## Discussion

The majority of clear cell renal cell carcinoma (ccRCC) tumors arise from sustained inactivating mutations in the VHL gene. The loss or silencing of VHL leads to the accumulation of HIFα, which in turn transcriptionally upregulates tumorigenic hypoxia-responsive genes. Significant progress has been made in targeting HIF2α and its downstream effector molecules, such as VEGF. However, a comprehensive understanding of the functional axis of HIF2α in the biology of metastatic ccRCC is crucial for achieving optimal therapeutic outcomes. In this study, we identified MCJ, a negative regulator within the mitochondrial respiratory chain, as a downstream molecule of HIF2α. Moreover, the activation of MCJ by HIF2α results in the disruption of mitochondrial function. The consequent abnormal accumulation of ROS promotes the stabilization and secretion of prolegumain, leading to oxidation at the His343 site and enhanced tumor metastasis.

The role of legumain in cancer is complex and multifaceted, encompassing its involvement in tumor progression, metastasis, and as a target for therapeutic intervention. Overexpression of legumain has been noted in a variety of solid tumors, yet its prognostic implications remain a subject of debate. In breast cancer, legumain fosters tumor cell proliferation and metastasis, while in glioblastoma, it is implicated in the inactivation of the tumor suppressor protein p53 and the cleavage of tropomodulin-3, contributing to cellular and genetic destabilization. In contrast, Fabbri et al. reported that elevated legumain expression is associated with a more favorable prognosis in renal cell carcinomas (RCCs) [26]. In the present study, we compared legumain levels in ccRCC tumor tissues and adjacent normal kidney tissues. Our findings revealed that the protein levels of legumain were elevated in tumor tissues compared to their paired normal counterparts. Intriguingly, the mature form of legumain was found to be significantly diminished in tumor tissues. Furthermore, the urinary levels of legumain were increased in ccRCC patients relative to controls and showed a positive correlation with the metastatic status of the tumor. These findings underscore the context-dependent role of legumain in tumors and suggest that the regulation of its cellular localization may contribute to the malignant progression of ccRCC.

Legumain, like other cysteine proteases, is initially synthesized as a precursor and is predominantly transported to endolysosomal compartments via the Golgi apparatus [21]. There is growing evidence that supports the non-intracellular distribution of legumain, such as on cell surfaces, within the extracellular matrix, and in extracellular vesicles [27]. Different environments support the molecular characteristics of legumain, including autoactivation, conformational stability, and enzymatic functions. An acidic pH and a reducing redox potential are required for the processing of prolegumain into the mature active ~36 kDa form [28]. Post-translational modifications, including ubiquitination and N-glycosylation, have been shown to be important for the transport, cellular localization, secretion, and stability of legumain [29, 30]. A recent study showed that dysregulation of the oxidation site accelerates intracellular legumain processing and tumor progression after H. pylori infection [18]. Here, our data demonstrate that the oxidation site His343, resulting from excess ROS accumulation, promotes the secretion of prolegumain in ccRCC cells, an effect that can be rescued by scavengers of ROS/mtROS or by reducing ROS production. Furthermore, we identified that secreted legumain exhibits enzymatic activity in the cleavage of proMMP-2. Extracellular legumain undergoes autoactivation in the acidic tumor microenvironment and participates in the remodeling of the ECM, either by directly hydrolyzing matrix components like fibronectin or by promoting the maturation of MMPs. In addition to fibronectin and MMP-2, the classical tumor suppressor p53 and the ECM signal receptor integrin aVb3 have been identified as substrate proteins of legumain [31, 32]. Although the exact substrate proteins contributing to the pro-metastatic role of extracellular legumain in the tumor microenvironment cannot be completely clarified, the secretion of prolegumain and its exhibition of endopeptidase activity aid in the escape of tumor cells and the establishment of the metastatic niche.

In this study, we identified MCJ as a downstream molecule of HIF2α in clear cell renal cell carcinoma (ccRCC). MCJ, an endogenous negative regulator of Complex I, is a transmembrane protein located in the inner mitochondrial membrane and is predominantly expressed in highly metabolic tissues, including the liver, heart, and kidney [24, 25, 33]. In a mouse model of nonalcoholic steatohepatitis, silencing MCJ effectively reduces liver lipid accumulation and fibrosis [34]. Renal tubular cells are highly differentiated cells characterized by a low metabolic rate and high energy consumption. Our data reveal that overexpression of MCJ is associated with more severe mitochondrial damage in ccRCC cells compared to control tubular cells. Downregulation of MCJ improves mitochondrial damage, increases ATP levels, and reduces the GSSG/GSH ratio and mtROS in ccRCC cells, suggesting an enhancement of mitochondrial function. The role of HIF2α in RCC is well established and recognized as an oncoprotein that is both sufficient and necessary to promote xenograft tumor growth [35, 36]. As a result of the accumulation and translocation of HIF-α factors into the nucleus, dimerization occurs with a constitutively expressed HIFβ-subunit. This complex transactivates various genes encoding molecules that are causally correlated with the development of ccRCC. These include angiogenic growth factors VEGFA and PDGFB, growth factor TGFα, cell-cycle regulator cyclin D1, the glucose transporter GLUT1, and the chemokine SDF and its receptor CXCR4 [37]. Here, identifying MCJ as a transcriptional target of HIF2α, which subsequently enhances legumain secretion, provides further insights into the role of HIF2α in the development of ccRCC.

## Conclusion

In summary, our findings suggest that HIF2α initiates the expression of MCJ, which in turn leads to increased legumain secretion in ccRCC. Clinically, the level of legumain in urine may serve as a potential early diagnostic biomarker for ccRCC. Moreover, targeting MCJ to inhibit legumain secretion or enhance the clearance of reactive oxygen species (ROS) presents promising therapeutic strategies for the management of metastatic ccRCC.

## Supplementary information

Supplementary figures and legends

Raw data-Gels and Blots images

## Data Availability

The datasets referenced to support the conclusions of this research are included in the article and its supplementary files.
