# Supplementary material for: HIF2α drives ccRCC metastasis through transcriptional activation of methylation-controlled J protein and enhanced prolegumain secretion
Source: Cell Death Dis. 2025 Feb 13;16(1):93. doi: 10.1038/s41419-025-07432-3 (PMC11825665; doi:10.1038/s41419-025-07432-3)
Supplement: Supplementary file 1 — Supplementary figures and legends [file 41419_2025_7432_MOESM1_ESM.pdf]

HIF2α Drives ccRCC Metastasis through Transcriptional Activation of Methylation-controlled J Protein and Enhanced Prolegumain Secretion

Tianyu Shen[1, #], Yu Su[1, #], Dekun Wang[1], Chuangxin Sun[2], Gang Li[2], Taoyu Hu[1], Haoxiang Pang[1], Xue Mi[1], Yuying Zhang[1], Shijing Yue[1], Zhujun Zhang[1], Xiaoyue Tan[1]✉

1. The School of Medicine, Nankai University; 94 Wei Jin Road, Tianjin, China
2. Department of Urology, Tianjin Institute of Urology, the 2nd Hospital of Tianjin Medical University, 23 Ping Jiang Road, Tianjin, China

# These authors contribute equally to this paper.

✉ Corresponding author: Xiaoyue Tan: Tel: (86) 13820857192; Mail: xiaoyuetan@nankai.edu.cn

Key words: Clear cell Renal Cell Carcinoma; Legumain; Hypoxia-inducible factor 2α; Methylation-controlled J; Remodeling of extracellular matrix

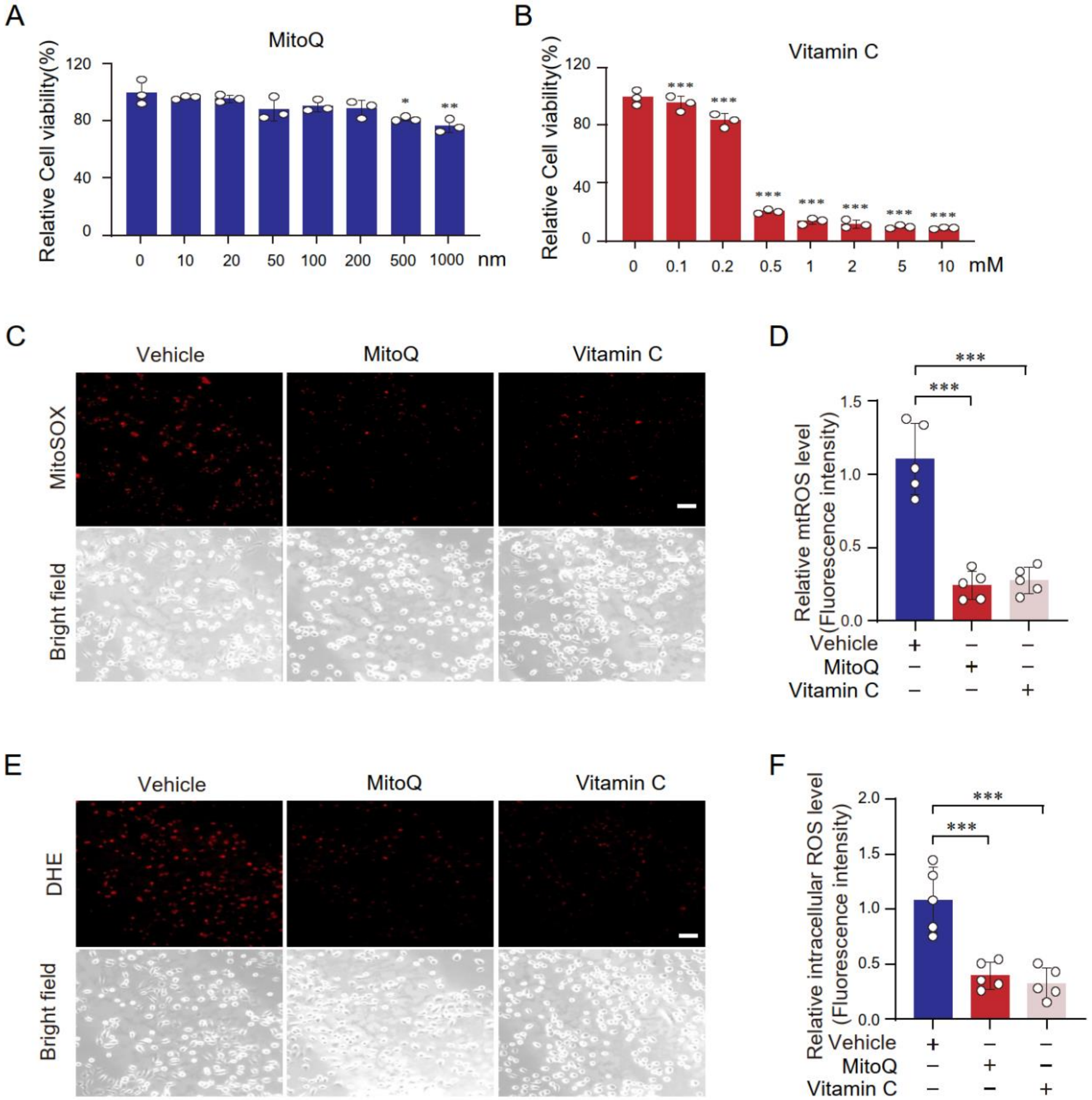

Supplementary Figure 1. Vitamin C or MitoQ reduces the level of ROS in 786-O cells. 786-O was treated with different concentrations of MitoQ and Vitamin C, respectively, for 24 h. (A & B) The CCK-8 assay was used to detect cell viability. (C-F) The DHE probe and MitoSOX probe were labeled to show intracellular total ROS and mitochondrial ROS, respectively. Fluorescence intensity was used as a statistical indicator. All data represent the mean ± SD. *, $P < 0.05$; **, $P < 0.01$; ***, $P < 0.001$.

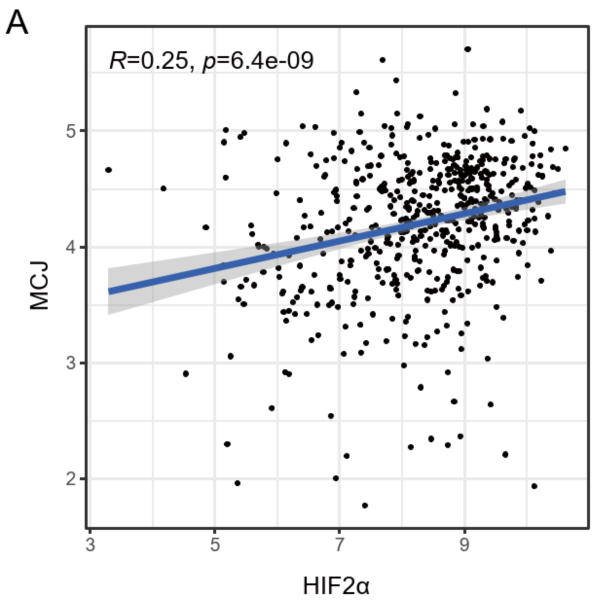

Supplementary Figure 2. The positive correlation between HIF2 and MCJ in ccRCCs. (A) The correlation coefficient between HIF2α and MCJ was calculated based on data from the TCGA database with Pearson R=0.25 and p=6.4e-09.

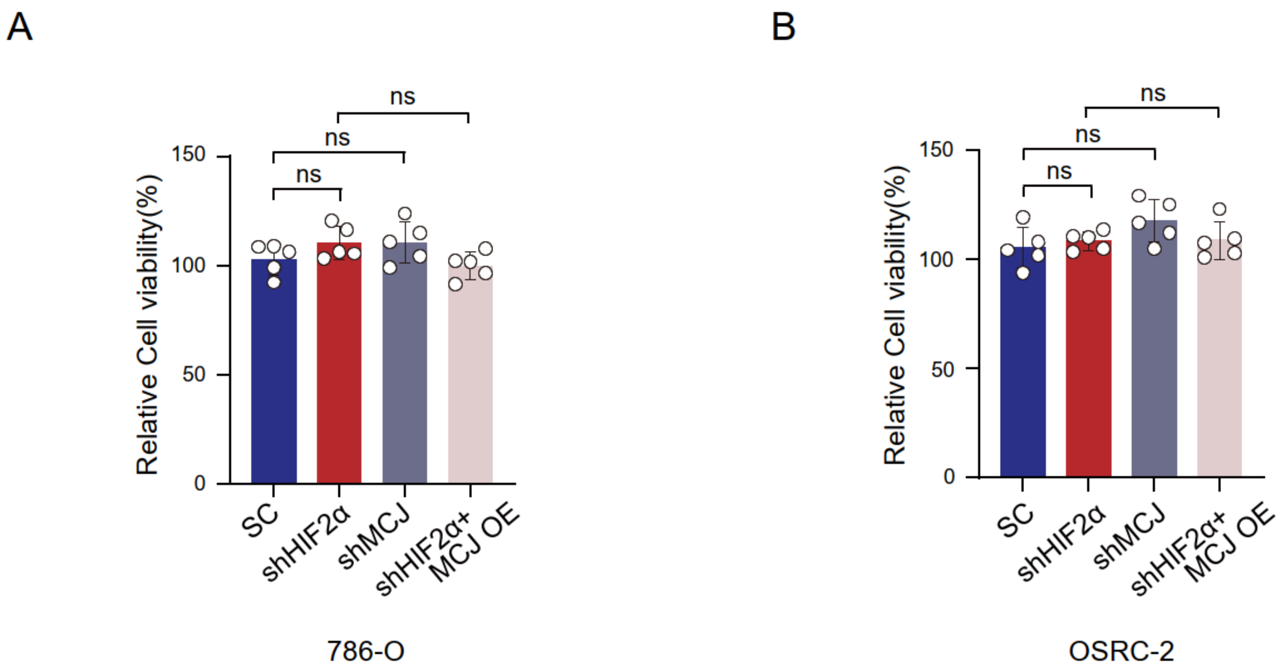

Supplementary Figure 3. No effect of conditioned medium on HUVEC cell proliferation after altering the levels of HIF2α or MCJ in tumor cells. Stable HIF2α and MCJ knockdown 786-O and OSRC-2 cell lines were established, and MCJ was transiently overexpressed in the stable HIF2α knockdown 786-O cells. Conditioned media of 786-O or OSRC-2 cells were collected and used to stimulate HUVEC. (A & B) The CCK-8 assay was used to detect the cell viability of HUVEC. All data represent the mean ± SD. ns, $P > 0.05$.
