# Supplementary material for: HIF2α drives ccRCC metastasis through transcriptional activation of methylation-controlled J protein and enhanced prolegumain secretion
Source: Cell Death Dis. 2025 Feb 13;16(1):93. doi: 10.1038/s41419-025-07432-3 (PMC11825665; doi:10.1038/s41419-025-07432-3)
Supplement: Supplementary file 2 — Raw data-Gels and Blots images [file 41419_2025_7432_MOESM2_ESM.pdf]

Fig 1B

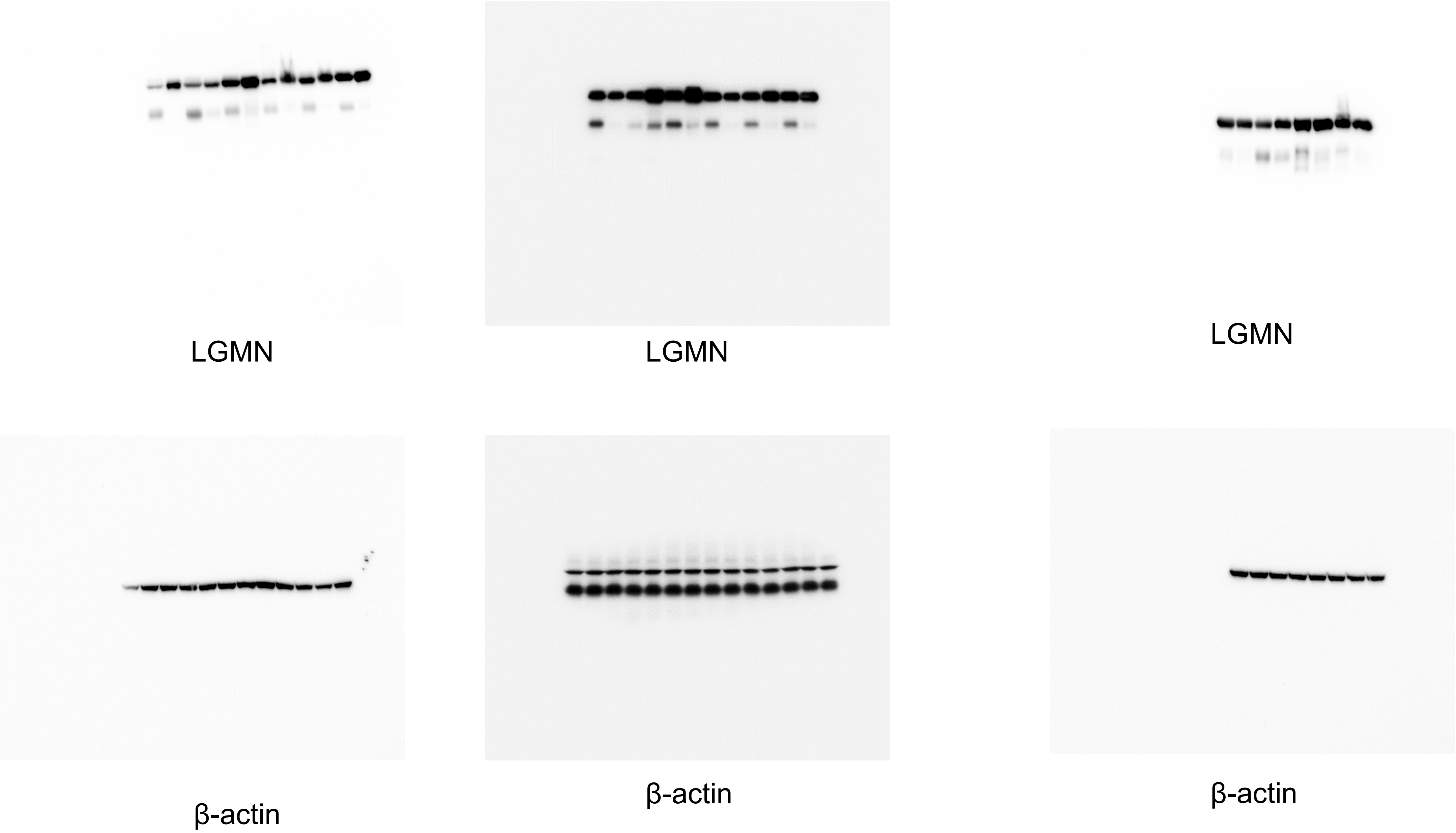

Fig 1E

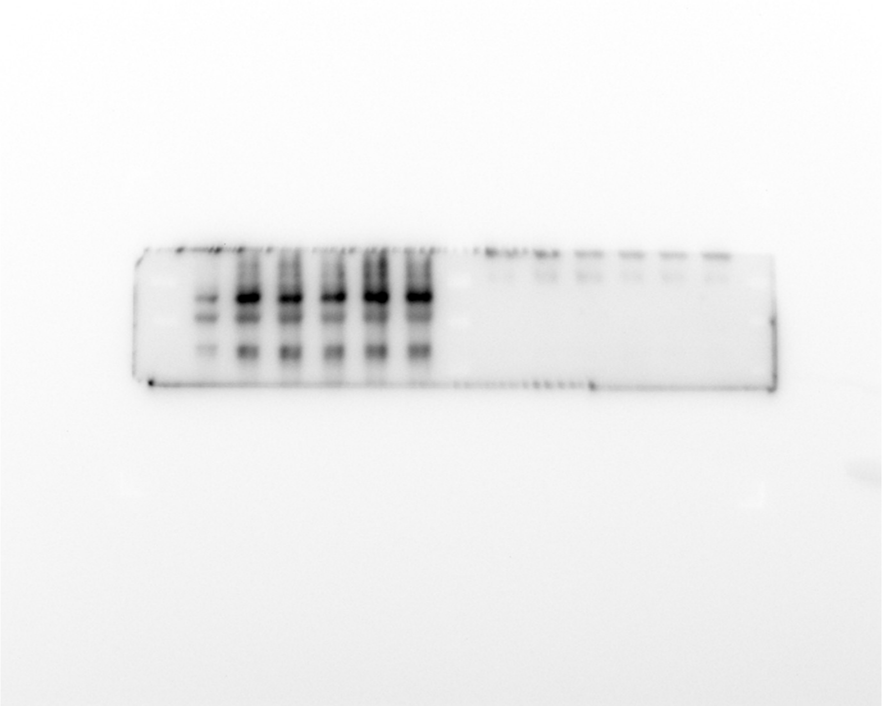

LGMN

Fig 1G

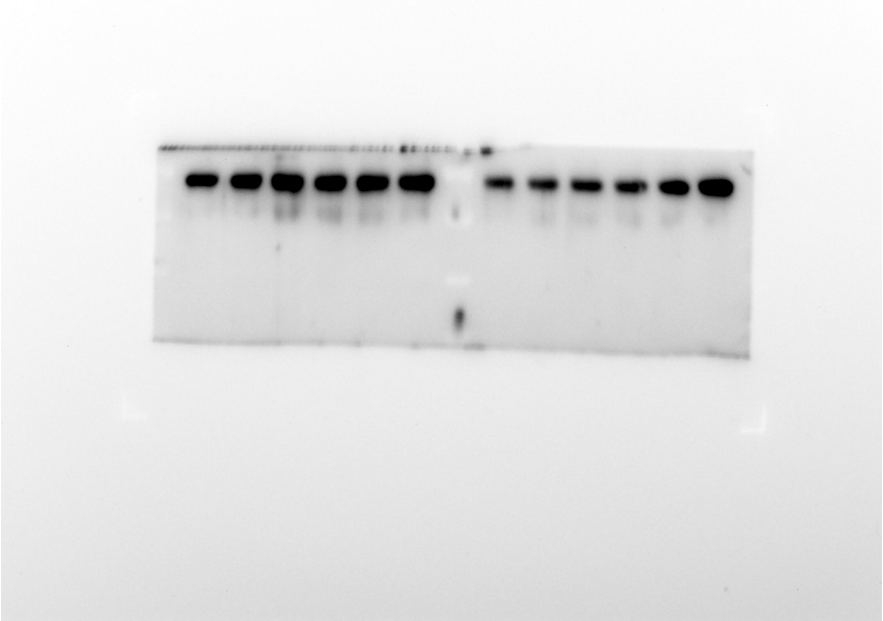

LGMN

Fig 2C

Cell lysate

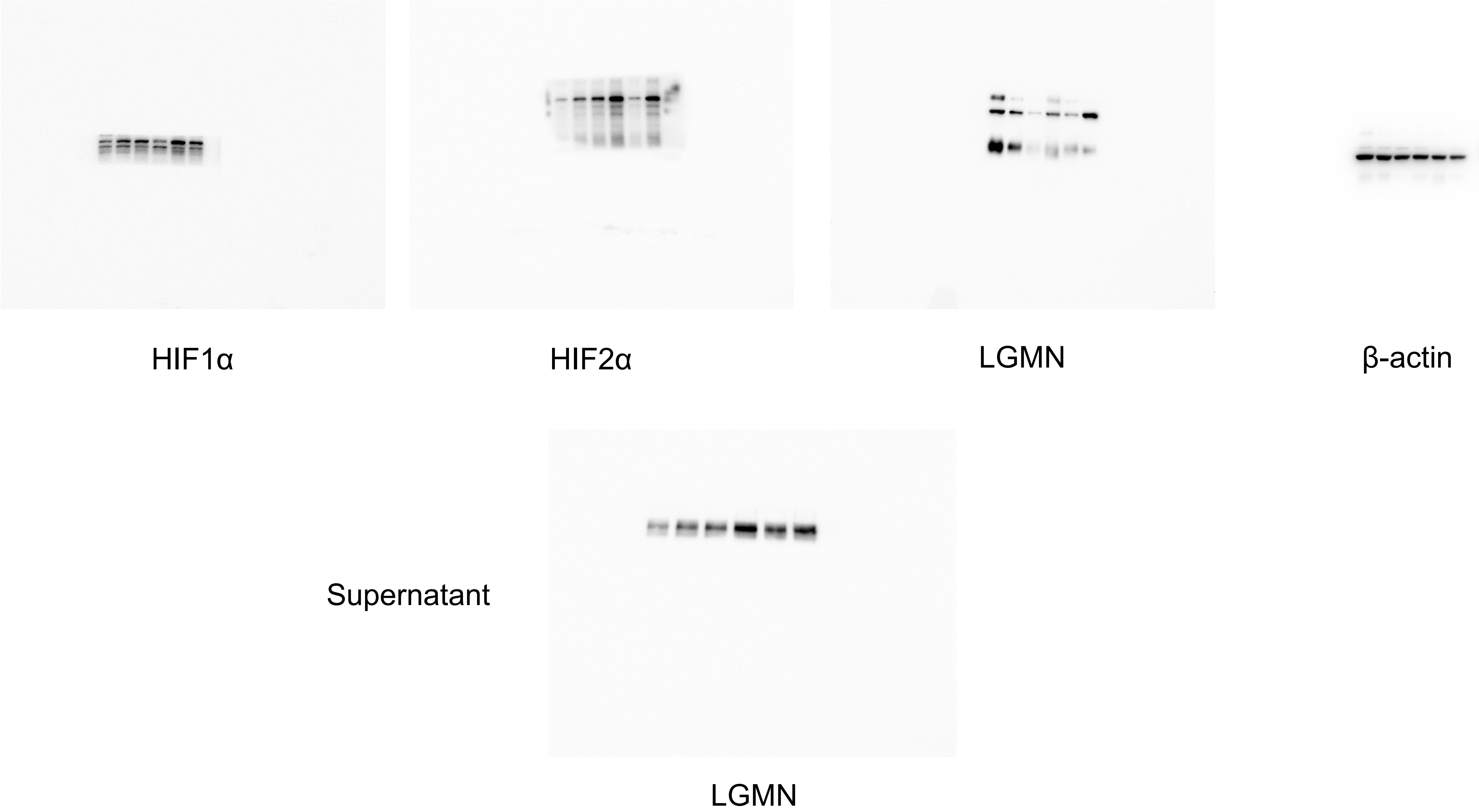

Fig 2E

786-O

Cell lysate

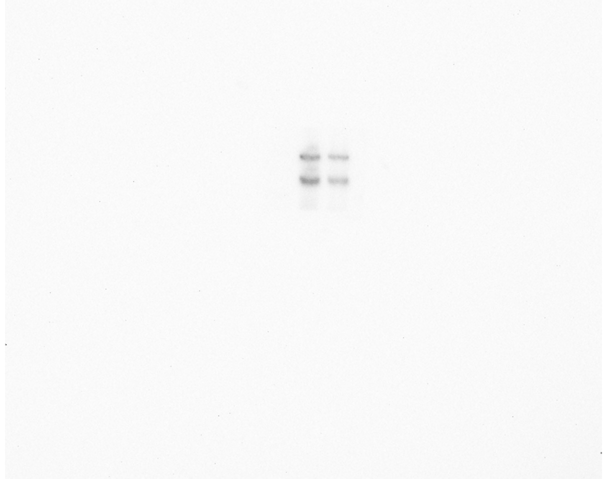

HIF2α

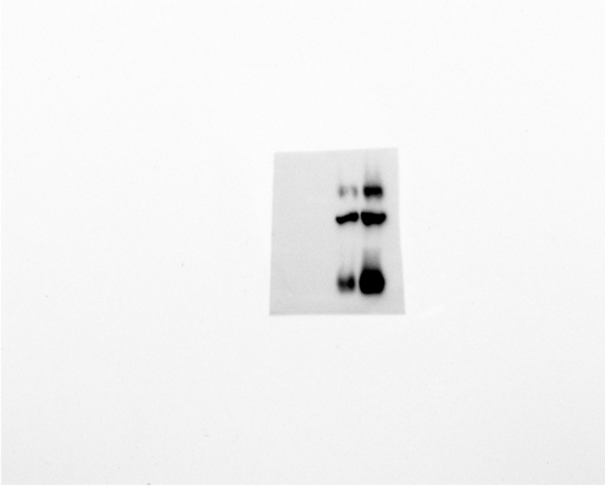

LGMN

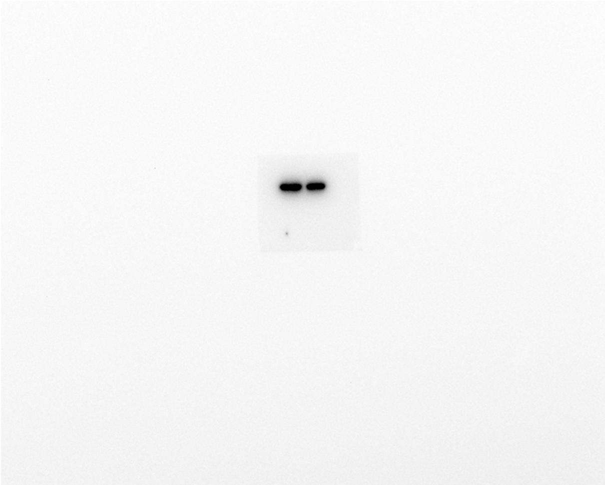

β-actin

Supernatant

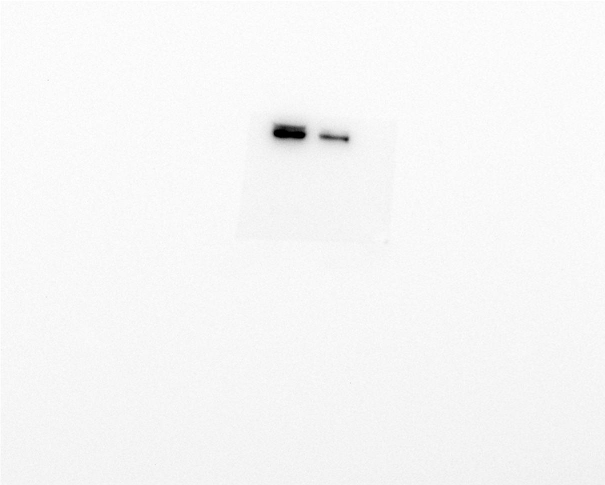

LGMN

OSRC-2

Cell lysate

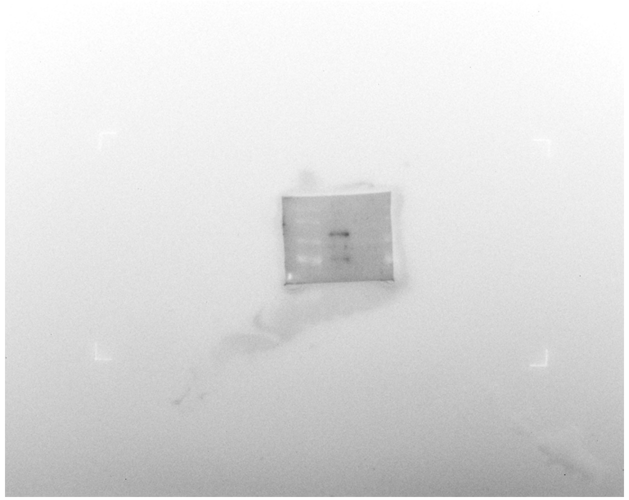

HIF2α

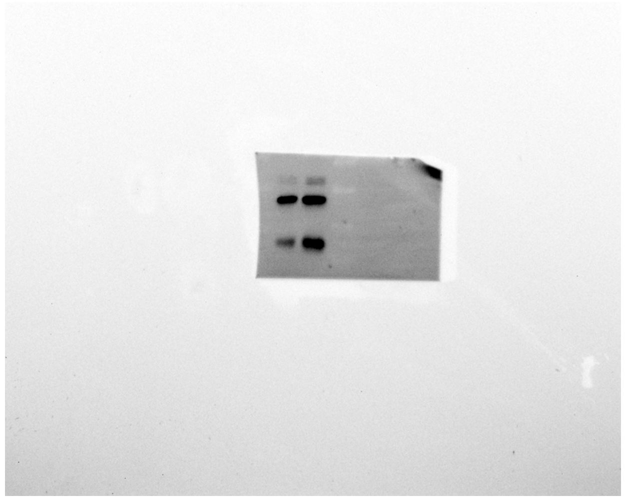

LGMN

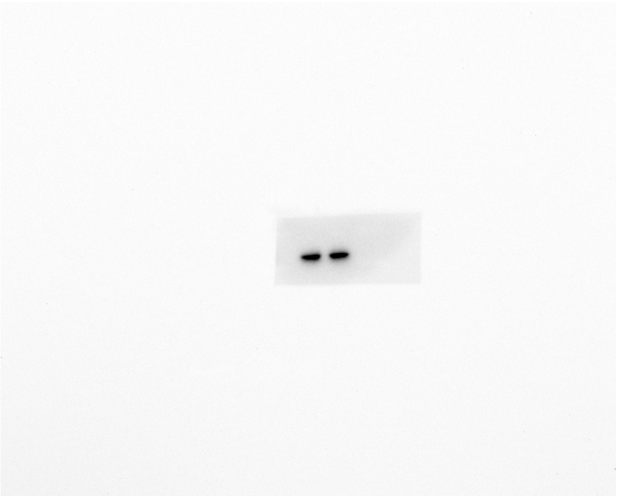

β-actin

Supernatant

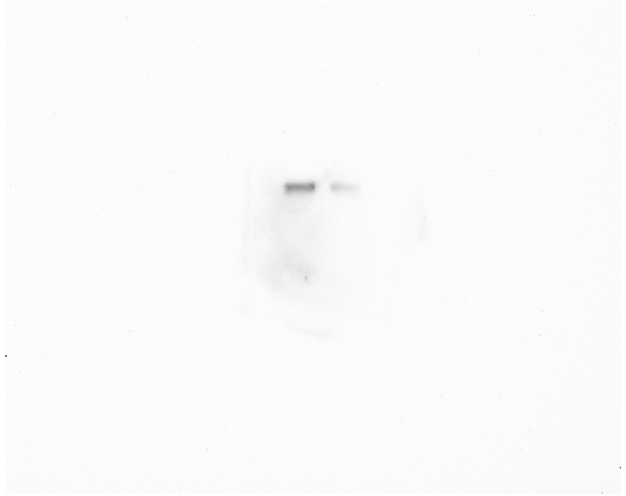

LGMN

Fig 3A

786-O

Cell lysate

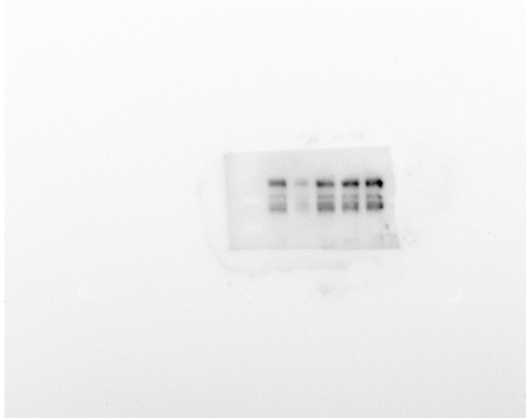

HIF2α

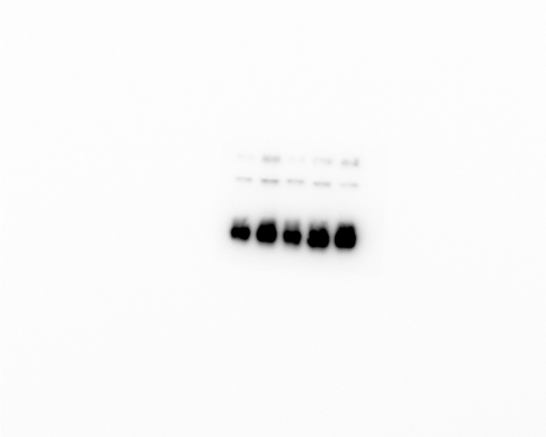

LGMN

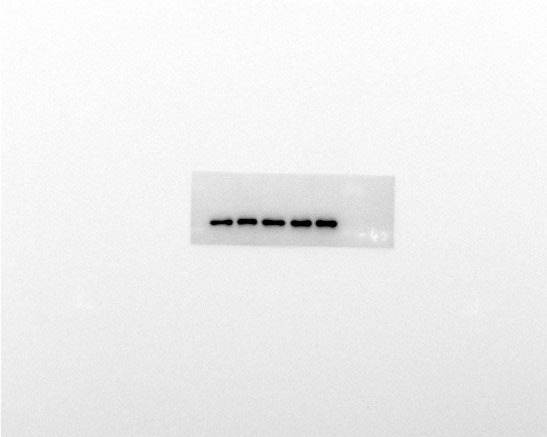

β-actin

Supernatant

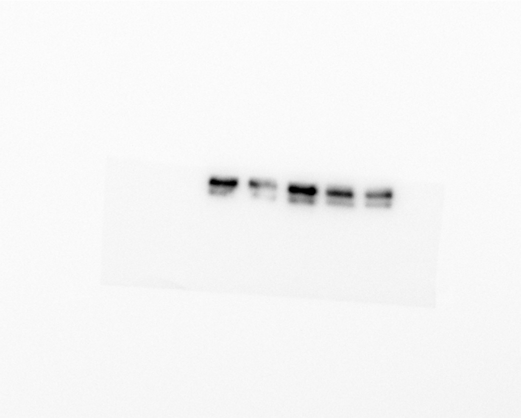

LGMN

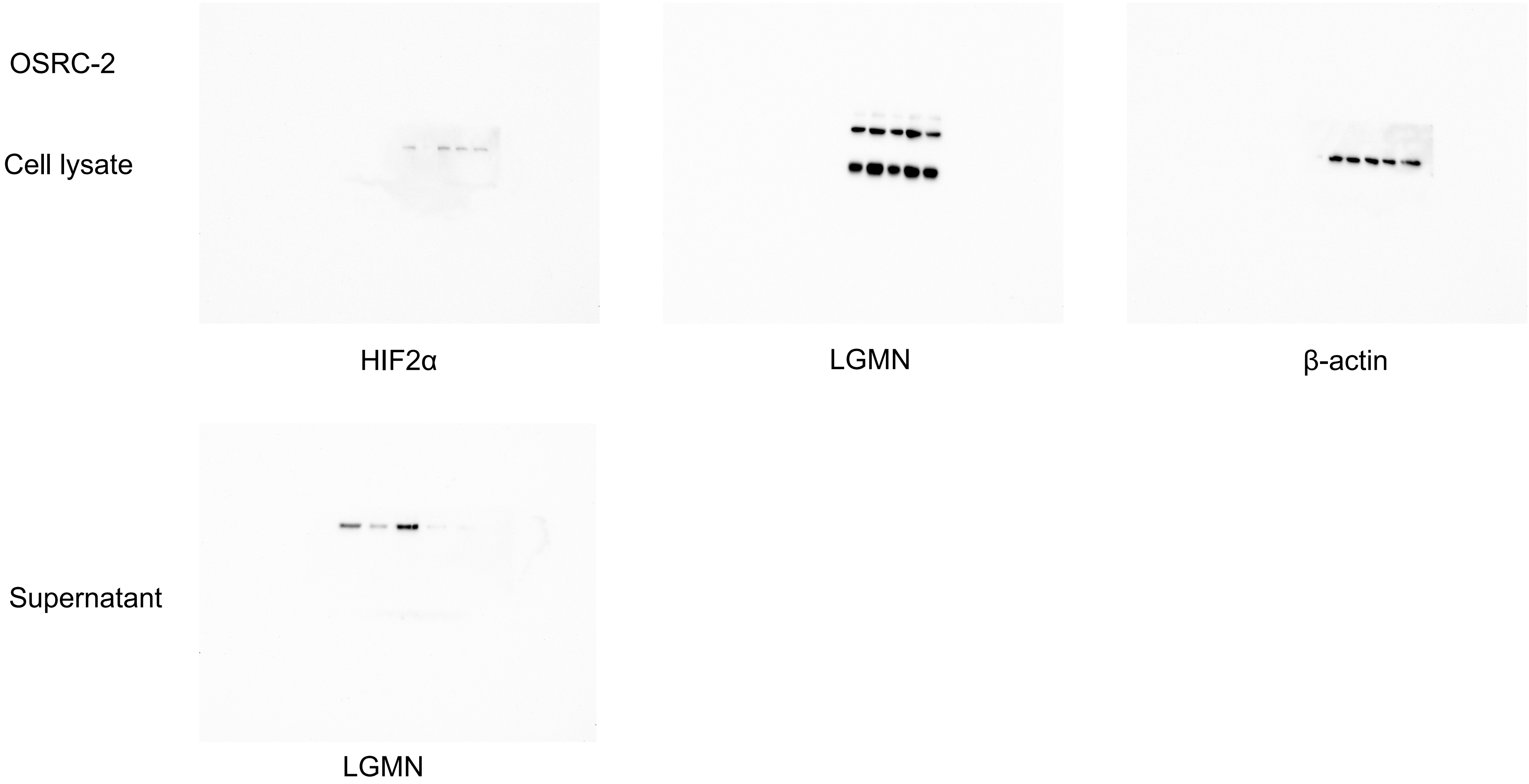
OSRC-2
Cell lysate
HIF2α
LGMN
β-actin
Supernatant
LGMN

Fig 3D

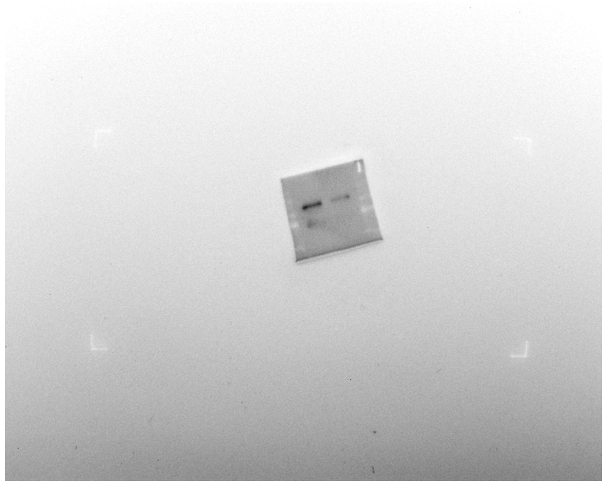

HIF2α

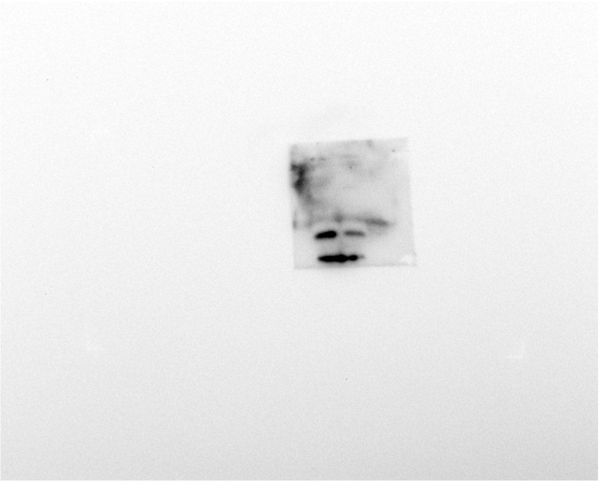

MCJ

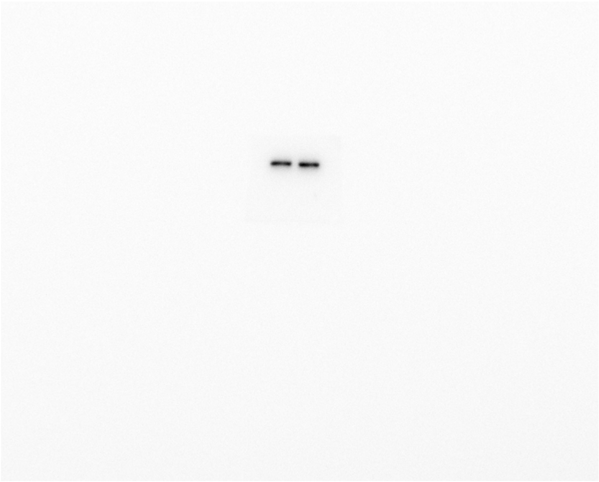

β-actin

Fig 3F

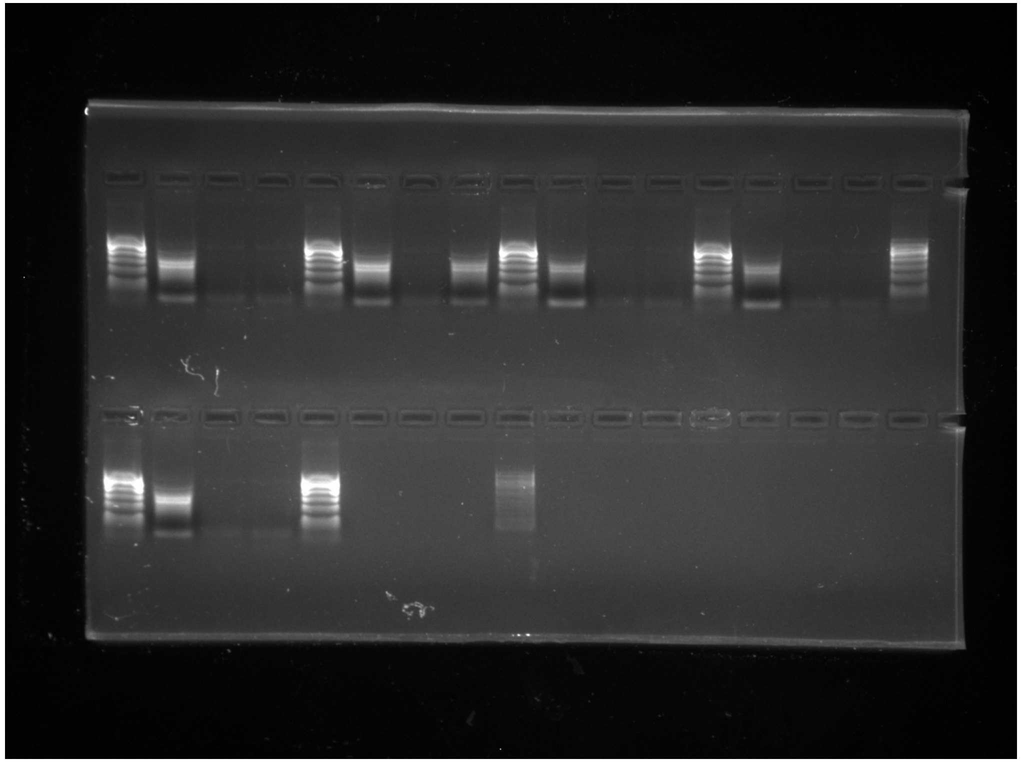

Fig 3G

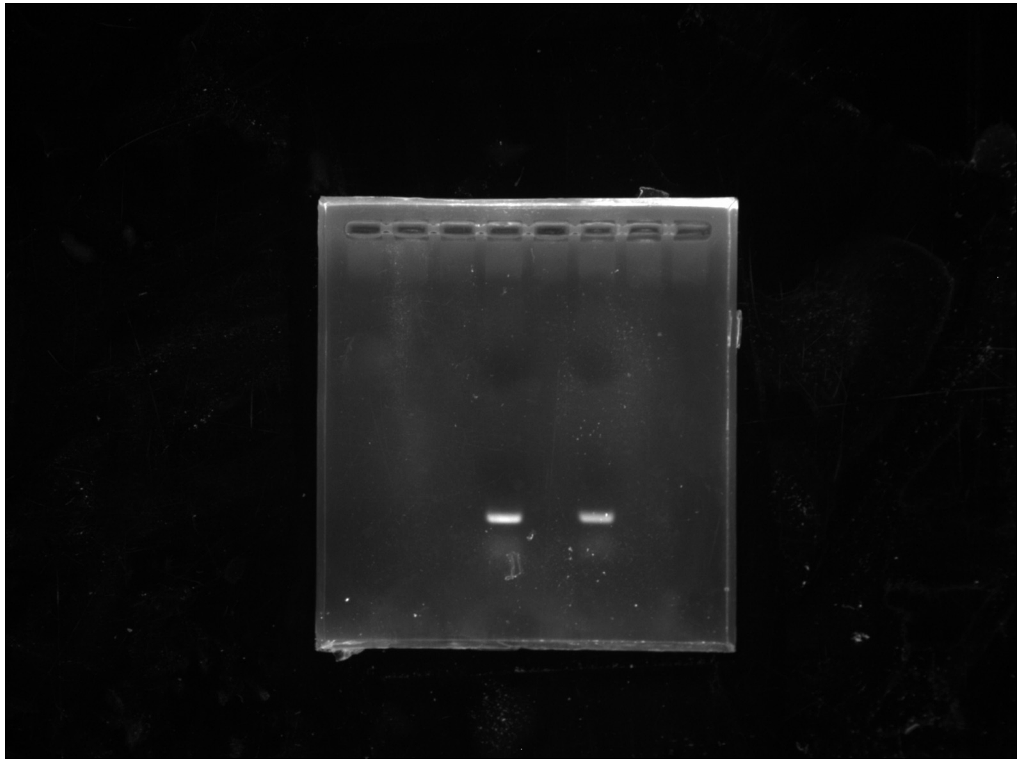

Fig 3I

Cell lysate

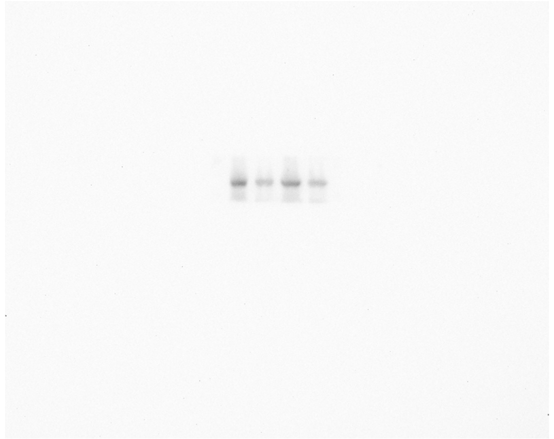

HIF2α

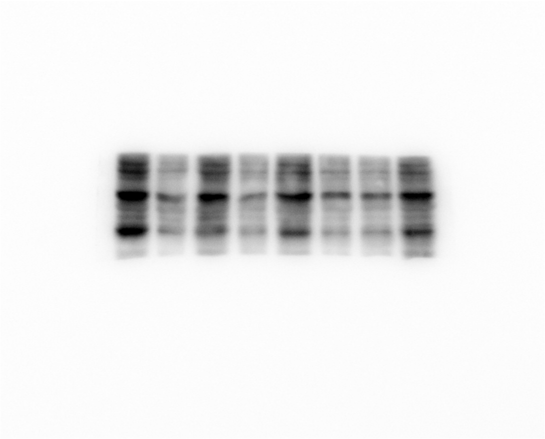

MCJ

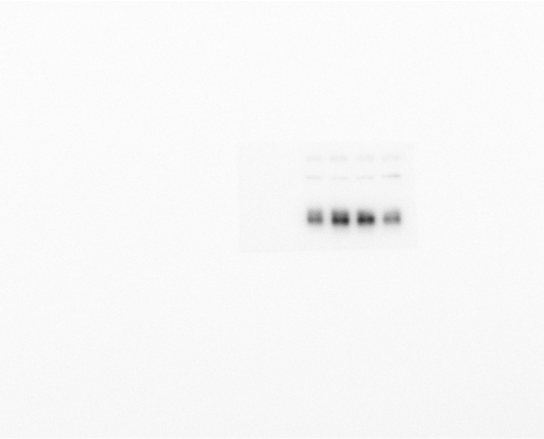

LGMN

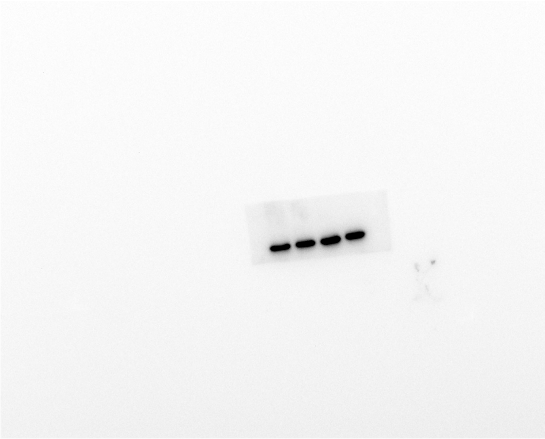

β-actin

Supernatant

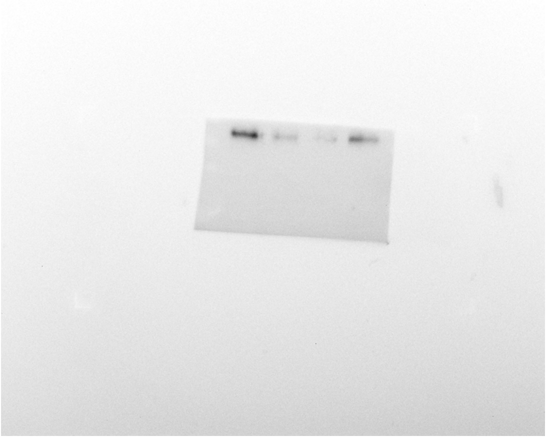

LGMN

Fig 5E

Cell lysate

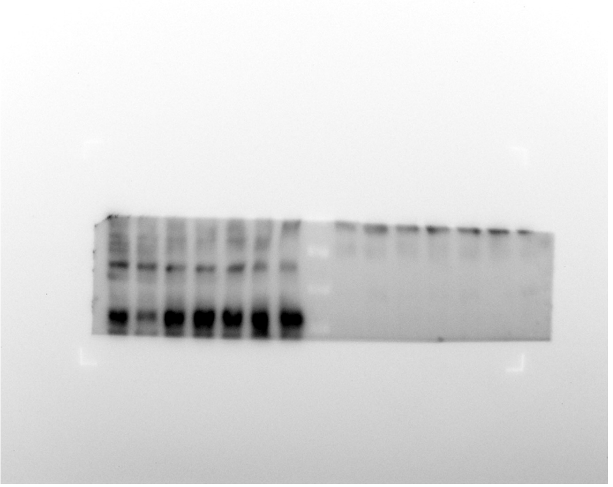

LGMN

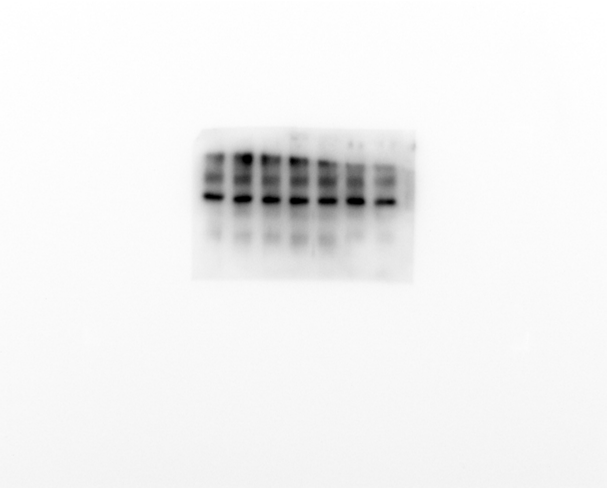

β-actin

Supernatant

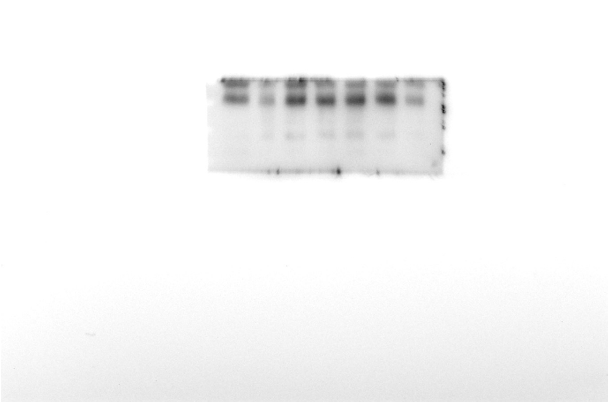

LGMN

Fig 6A

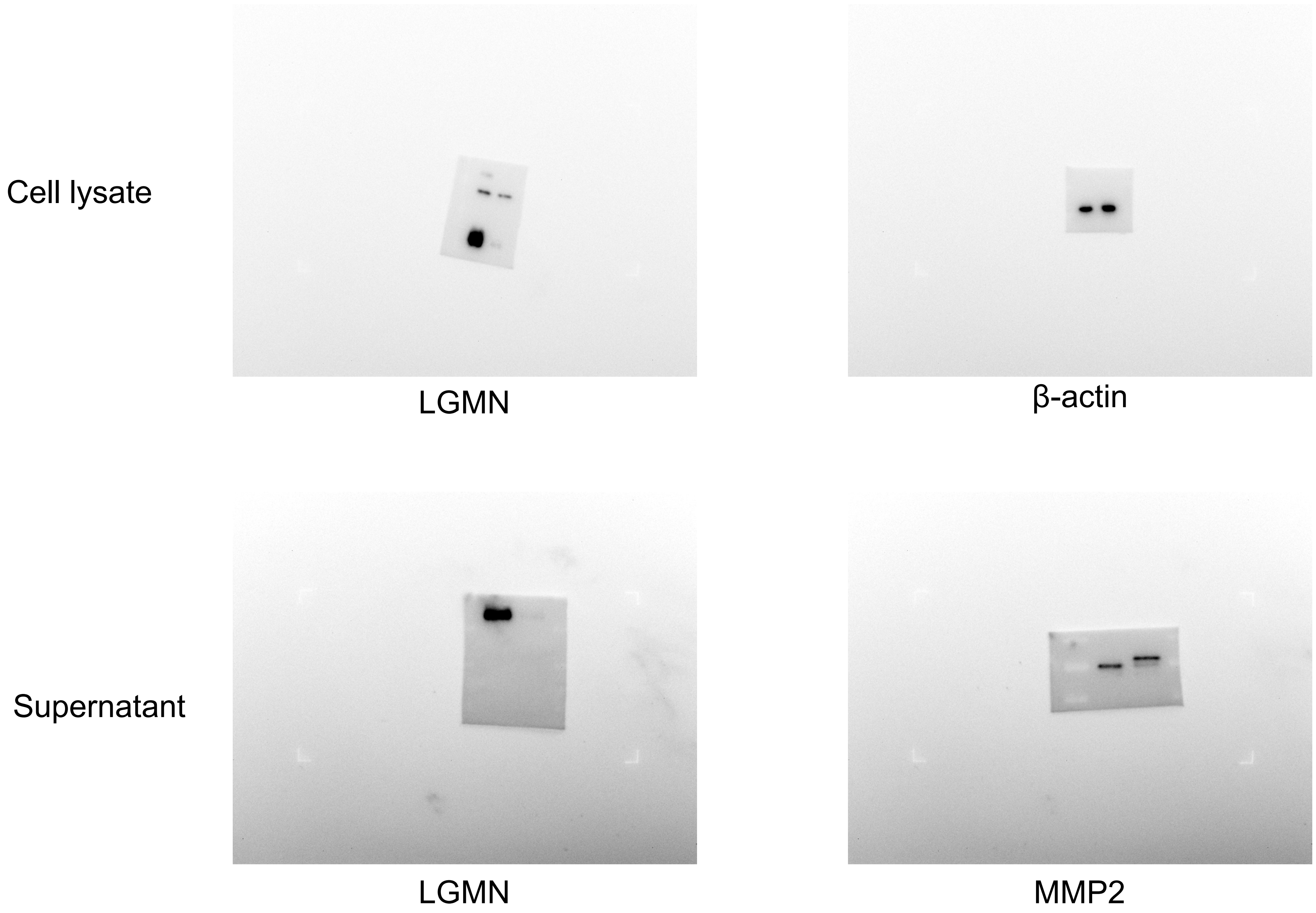

Fig 6B

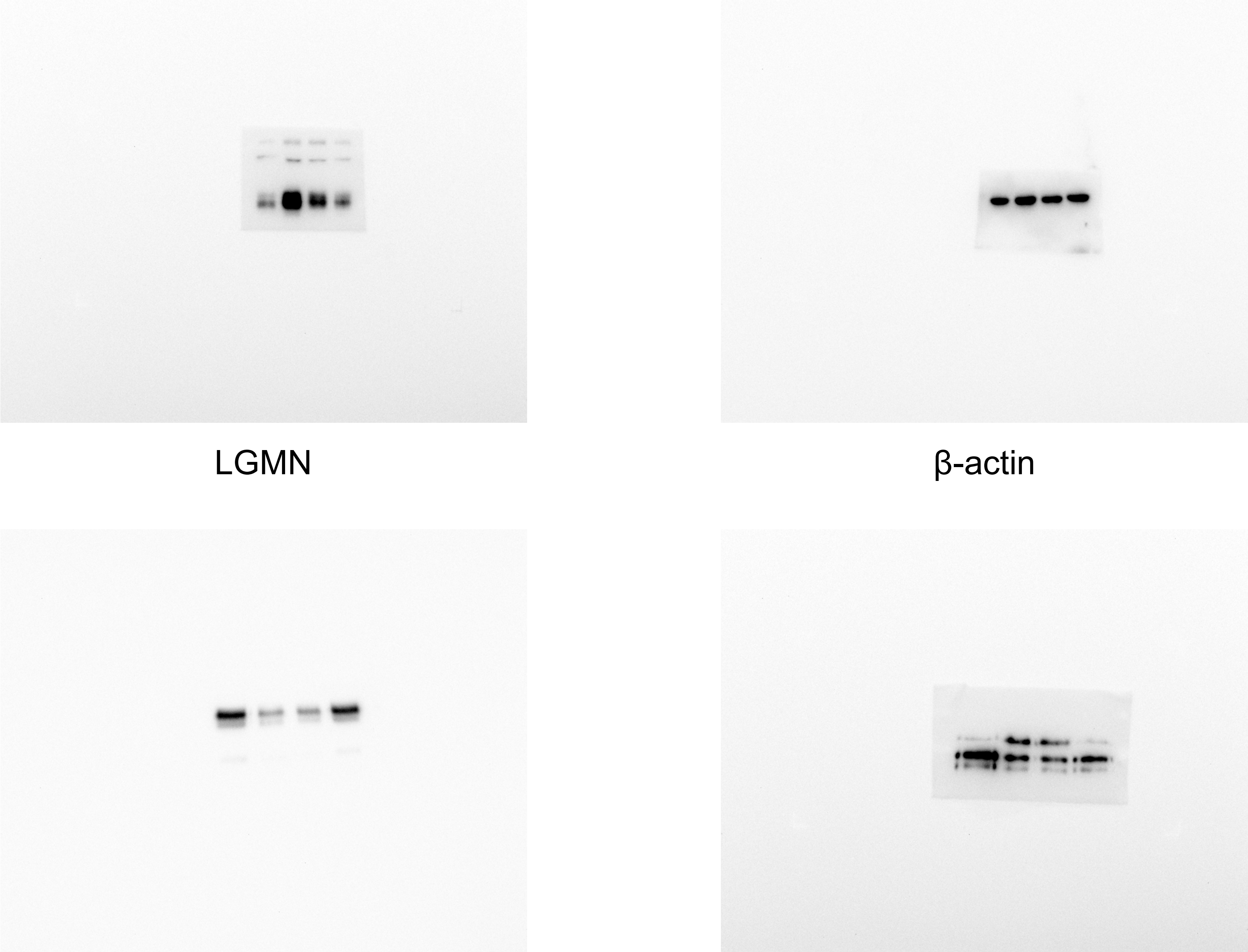

Cell lysate: LGMN, β-actin

Supernatant: LGMN, MMP2

Fig 6C

Cell lysate

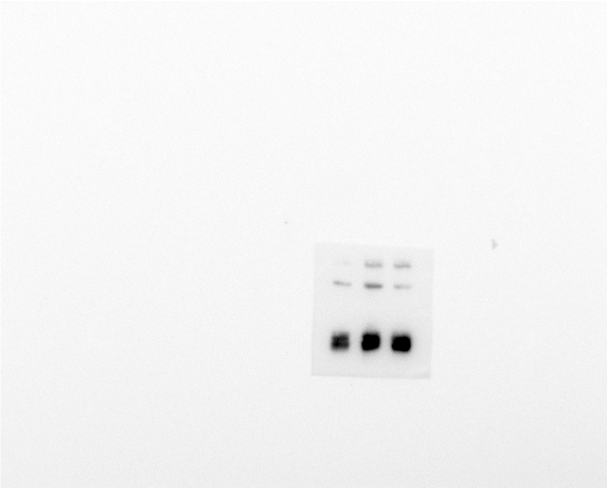

LGMN

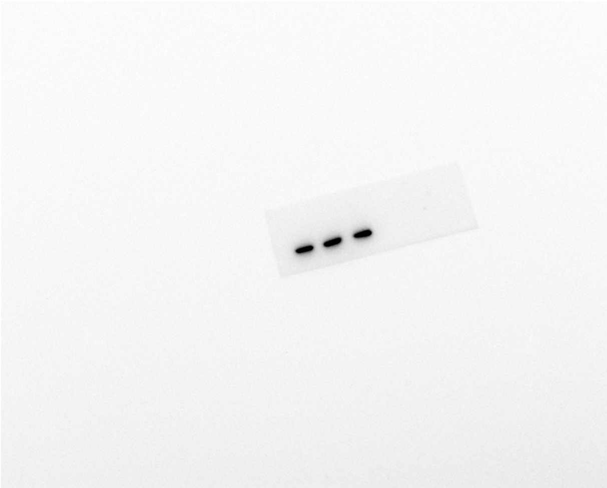

β-actin

Supernatant

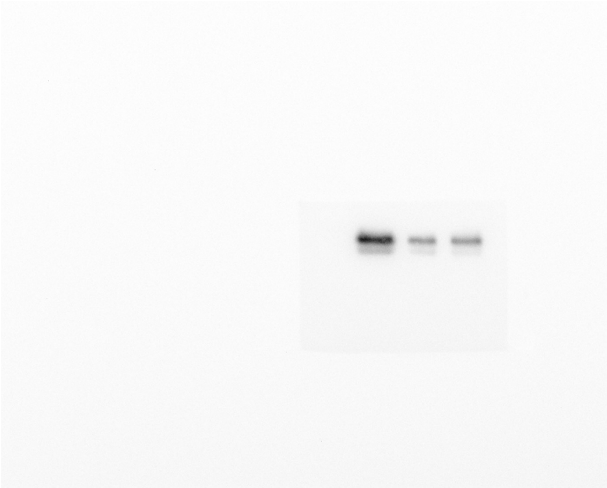

LGMN

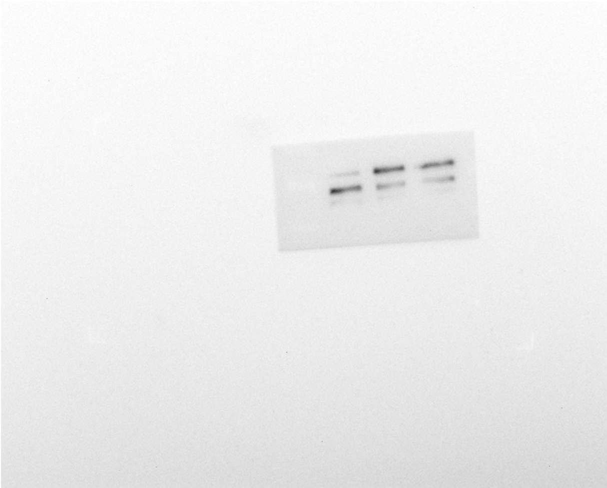

MMP2

Fig 7N

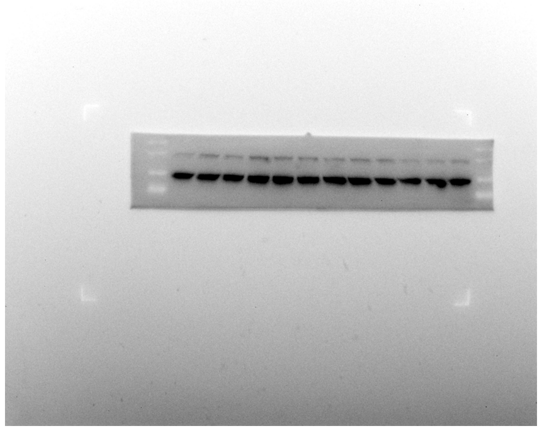

HIF2α

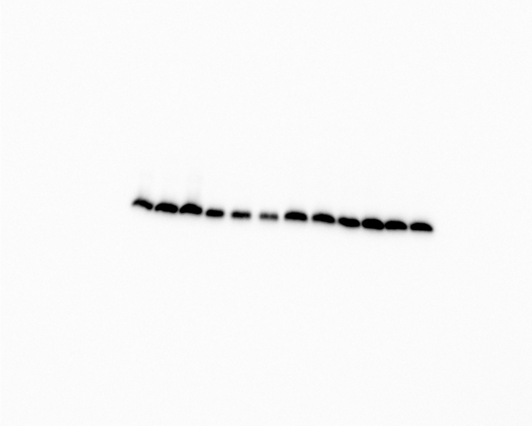

MCJ

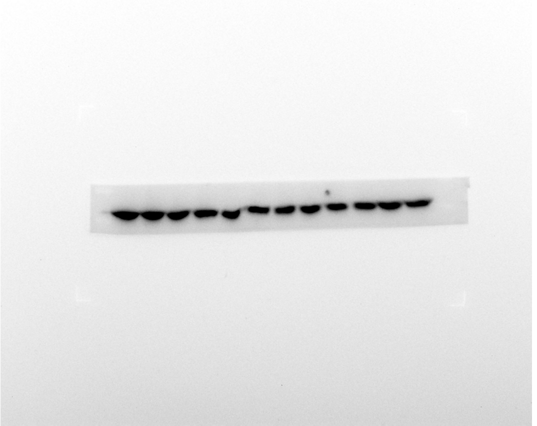

β-actin
